# Positively Selected Sites in Cetacean Myoglobins Contribute to Protein Stability

**DOI:** 10.1371/journal.pcbi.1002929

**Published:** 2013-03-07

**Authors:** Pouria Dasmeh, Adrian W. R. Serohijos, Kasper P. Kepp, Eugene I. Shakhnovich

**Affiliations:** 1 Technical University of Denmark, DTU Chemistry, Kongens Lyngby, Denmark; 2 Department of Chemistry and Chemical Biology, Harvard University, Cambridge, Massachusetts, United States of America; Tel Aviv University, Israel

## Abstract

Since divergence ∼50 Ma ago from their terrestrial ancestors, cetaceans underwent a series of adaptations such as a ∼10–20 fold increase in myoglobin (Mb) concentration in skeletal muscle, critical for increasing oxygen storage capacity and prolonging dive time. Whereas the O_2_-binding affinity of Mbs is not significantly different among mammals (with typical oxygenation constants of ∼0.8–1.2 µM^−1^), folding stabilities of cetacean Mbs are ∼2–4 kcal/mol higher than for terrestrial Mbs. Using ancestral sequence reconstruction, maximum likelihood and Bayesian tests to describe the evolution of cetacean Mbs, and experimentally calibrated computation of stability effects of mutations, we observe accelerated evolution in cetaceans and identify seven positively selected sites in Mb. Overall, these sites contribute to Mb stabilization with a conditional probability of 0.8. We observe a correlation between Mb folding stability and protein abundance, suggesting that a selection pressure for stability acts proportionally to higher expression. We also identify a major divergence event leading to the common ancestor of whales, during which major stabilization occurred. Most of the positively selected sites that occur later act against other destabilizing mutations to maintain stability across the clade, except for the shallow divers, where late stability relaxation occurs, probably due to the shorter aerobic dive limits of these species. The three main positively selected sites 66, 5, and 35 undergo changes that favor hydrophobic folding, structural integrity, and intra-helical hydrogen bonds.

## Author Summary

In this work, we identify positive selection in cetacean myoglobins and an early, significant divergence event. While O_2_-binding is nearly unchanged, positive selection acts to introduce and later maintain stability. Stability correlates with abundance across the species, supporting that selection for increased stability concurred with the known 10–20 fold increase in myoglobin abundance of cetaceans relative to terrestrial mammals, which itself resulted from speciation towards longer dive lengths of the animals. We suggest that this selection acted to keep constant the otherwise increasing number of unfolded Mb. Altogether, this work for the first time links protein phenotype (stability and abundance) in a specific, real protein to organism-level evolution and fitness of mammals.

## Introduction

Upon adapting to the aquatic environment, marine mammals acquired features that improved their diving skills such as increased blood volume and hematocrit, efficient modes of locomotion (stroke-and-glide swimming) [1], [2] and ∼10–20 times higher myoglobin (Mb) concentration (*C_Mb_*) in the skeletal muscles contributing substantially to total body oxygen stores and aerobic dive limits [3], [4]. Using an integrated Krogh model of the muscle cell, models of convective oxygen transport and aerobic dive limit (ADL), and thermodynamics of O_2_-binding, we recently showed that wild-type (WT) Mb is more efficient than mutants under severely hypoxic conditions, whereas low-affinity mutants are in fact better transporters at intermediate oxygen pressure [5]. Moreover, while many sites do not affect O_2_-binding, conserved WT Mb traits are critical for prolonging the ADL of the animals: As the extreme example, mutating the distal His-64 residue can reduce the ADL by up to 14 minutes under routine dive conditions, and *C_Mb_* almost linearly extends the ADL *ceteris paribus*, explaining the extreme increase in *C_Mb_* occurring in the cetaceans [6].

Despite the intense research into the structure, function and physiological role of Mb [5]–[9], the evolution of Mb is not well understood [7]. Several studies have suggested that Mb is under a selection pressure for its function and structural integrity [7], [10]–[11]. Based on amino acid chemical properties and comparative studies of known Mb sequences, some form of selection has been suggested in the evolution of mammalian Mb to favor retention of the conformational structure [10]. Moreover, it has been shown that variable sites in cetacean Mbs are fewer in number but more prone to change than primate Mbs suggesting a probable shift in the function of Mb in cetaceans [11]. However, it is still unclear what drives Mb evolution, as are the specific sites potentially under positive selection and the changes in phenotype they might introduce.

Mb is a relatively conserved protein in all mammals [12]. In a sequence alignment of Sperm whale, Pig, Bovine, Dog, Sheep, Horse and Human Mb, 107 out of 153 residues, including those essential for O_2_ binding, are identical (See Text S1). Also, Mb oxygen affinity is nearly the same (K_O2_≈0.8–1.2 µM^−1^) for mammalian species. This observation is probably due to the “reversible binding” requirement of molecular O_2_ to Mb [13] at a given oxygen pressure, P_O2_, which strongly constrains oxygen binding thermodynamics across mammalian cells [5]. Despite similar K_O2_, another protein phenotype, the folding stability (i.e. the free energy of folding the protein, Δ*G_folding_* = *G_folded_−G_unfolded_*), is systematically higher in marine mammals compared to their terrestrial counterparts [14]. In a study of mammalian apoMbs, sperm whale apoMb was found to be ∼2.5 kcal/mol more stable than horse apoMb [15]. The stability difference can reach up to ∼4.5 kcal/mol when goose-beaked whale is compared to pig [16].

In this work, using current Bayesian methods to detect selection and a physical force field to compute the stability of single-point mutations, we first identify specific residues under positive selection in the cetacean clade and find that the evolution rate is substantially higher in cetacean Mbs compared to terrestrials. Second, we find that mutations in positively selected sites overall contribute to maintaining stability. Third, using ancestral state reconstruction, we demonstrate that most stabilization occurred during the divergence of cetaceans from the terrestrials. Furthermore, we observe a correlation between Mb folding stability and its abundance across species, further confirming that Mb stabilization is selected for in proportion to protein abundance. Thus, the higher Mb abundance required by speciation of cetacean seem to be accompanied by a larger selection pressure to preserve stability, possibly to reduce the copy number of misfolded Mb in the cell, which is a suggested universal selection pressure for highly expressed proteins [17].

## Results/Discussion

### Phylogenetics

**Figure 1:**
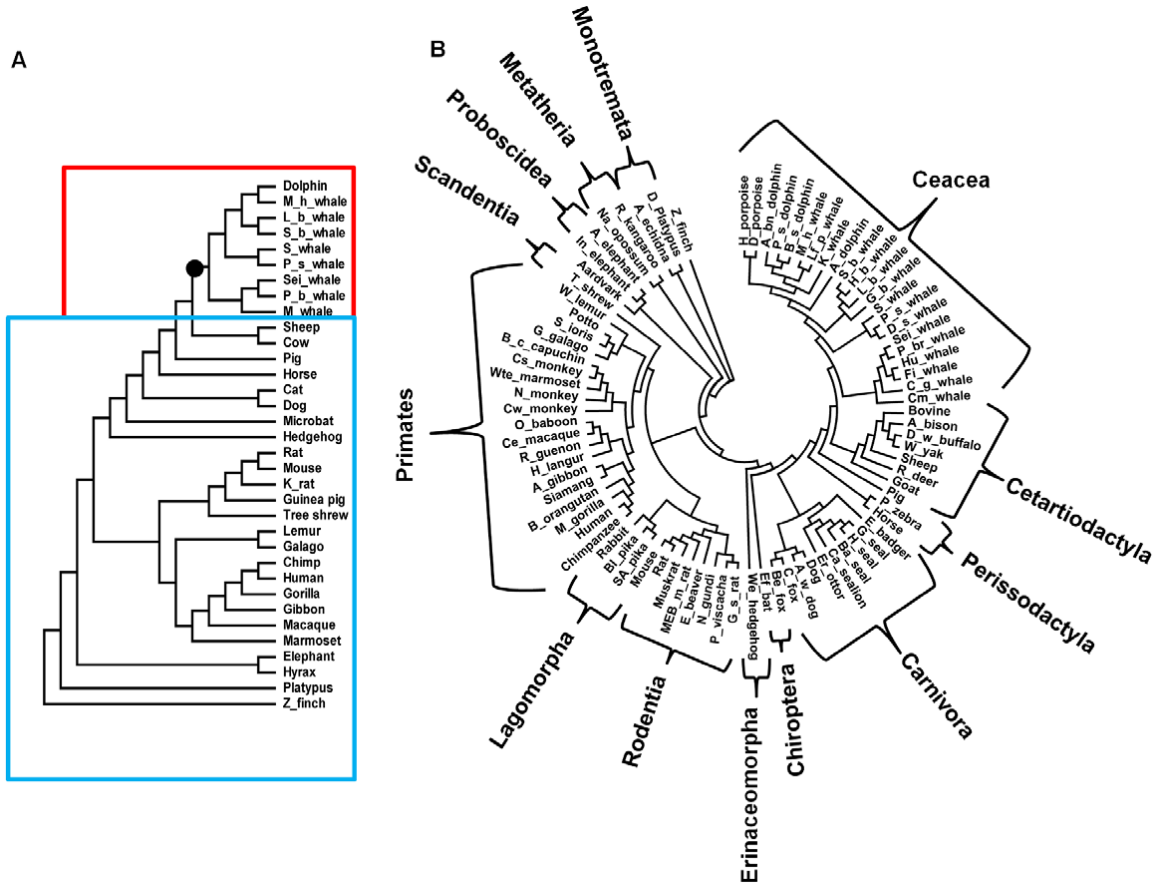
The mammalian phylogenetic tree constructed from A) nucleotide sequences and B) amino acid sequences. The smaller tree A was used in maximum likelihood tests for adaptive evolution while the tree B was explicitly used for ancestral state reconstruction. The best evolutionary model with the lowest BIC score was Tamura-Nei92 with transition/transversion bias, R = 1.66 in A and Dayhoff in B. Both models allow among-site-rate-variation sampled from a discrete gamma distribution with four categories and shape parameters 0.33 and 0.46 for nucleotide and amino acid sequences respectively. The phylogeny A is divided into two groups of cetaceans (shown in red) and terrestrial mammals (shown in blue) to test the non-uniformity of molecular clock across different lineages and sites. The branch leading to cetaceans is shown with a black circle in Figure 1A.

The available mammalian Mb sequences were divided into two datasets: 33 nucleotide sequences of mammalian Mbs were used to construct a phylogenetic tree used for evolutionary analysis with codon models (Figure 1A). To infer ancestral states with highest possible accuracy, a larger tree was also constructed from the substantially larger number (82) of available *amino acid* sequences of mammalian Mbs (Figure 1B). For both phylogenies, Zebra finch was the outgroup, cetaceans were divided into two major suborders, Mysticeti (minke whale and sei whale) and Odontoceti (sperm whales, beaked whales, dolphins, and porpoises), and all the branching patterns followed the known mammalian organism tree with order-specific patterns in primates, rodents, carnivore, cetardiodactylans, and cetaceans [18]–[26]. The accession numbers of all sequences used in this work, as well as full sequences of relevant ancestors are shown in Text S1.

The sequence of ancestral cetacean Mb was inferred from the available mammalian Mb sequences within all orders using the consensus mammalian species tree. Mb sequences from rodents and primates have minor effects on the most probable inferred ancestral sequence of cetacean Mb (see Text S1 for details).

### Detection of positive selection

To test for positive selection, we used codon-based models of nucleotide substitutions to estimate the rate of nonsynonymous to synonymous mutations, *dN/dS*, across different sites and branches of the mammalian phylogeny [27]. Also, all mutations were studied using the FoldX force field [28]–[30] to investigate whether the sites under selection in some way contribute to the stability phenotype of the Mbs (See Methods section for details).

Table 1 presents a comparison of the nested M0 (i.e. one *dN/dS* for all lineages) and FR (i.e. one *dN/dS* for each branch) models for both terrestrial and marine mammals. In the cetacean clade, the likelihood ratio test (LRT) gives a non-significant result of relatively similar ω ratios across the species. We also constrained ω to be the same in the whole cetacean clade (ω_1_) and different for the rest of the mammals (ω_0_). LRT is significant when it is compared with the one-ratio test with P-value<10^−16^. For ∼26% of sites in Mb, ω_1_ = 0.43 and ω_0_ = 0.19, testifying to a significantly higher evolution rate in cetaceans. As a further support, a higher rate of evolution was also observed in the whole-gene *dN/dS* comparison of cetaceans (Table 2) and primates (Table 3). The null hypothesis of two sets of *dN/dS* in primate and cetacean Mbs being similar is strongly rejected with the P-value of ∼1.33×10^−16^ using the two-sample t-test.

**Table 1:** Log likelihood values and parameter estimates of the site models, and branch-site models.

Clades	Model	ln L	Estimates of parameters	2Δl	P-value	Positively selected sites (BEB: P(ω>1)>0.50)^a^
**Cetacea**	M0 (one ratio)	−1241.82	ω_0_ = 0.1980			
	Free ratio	−1236.39	See Text S1	(M0 vs. Free ratio) 10.86	0.69	-
	**Site models**					
	M1a	−1251.18	p_0_ = 0.83845, p_1_ = 0.16155, ω_0_ = 0.02688, ω_1_ = 1			-
	M2a	−1248.47	p_0_ = 0.84199, p_1_ = 0.14878, p_2_ = 0.00922, ω_0_ = 0.03212, ω_1_ = 1.00000, ω_2_ = 4.91963	(M1a vs M2a) 5.42	0.06	**5**, 22, 35, **51**, **66**, 121, **129**
	M7	−1251.39	p = 0.06085 q = 0.29213			-
	M8	−1247.47	p_0_ = 0.98777, p = 0.11682, q = 0.66881, p_1_ = 0.01223, ω = 4.33010	(M7 vs. M8) 7.84	0.019	**5**, 22, 35, **51**, **66**, **121**, **129**
	M8fix	−1251.06	p_0_ = 0.86441, p = 0.11615, q = 2.08136, p_1_ = 0.13559, ω = 1.00000	(M8fix vs M8) 7.18	7.37×10^−3^	-
**Terrestrial mammals**	M0 (one ratio)	−4499.91	ω_0_ = 0.1062			-
	Free ratio	−4469.29	See Text S1	(M0 vs. Free ratio) 61.24	0.065	-
**Mammals**	M0 (one ratio)	−4926.63	ω_0_ = 0.08			-
	Free ratio	−4872.64	See Text S1	(M0 vs. Free ratio) 107.98	4.8×10^−4^	-
	**Site models**					
	M1a	−4646.77	p_0_ = 0.88207, p_1_ = 0.11793, ω_0_ = 0.05590, ω_1_ = 1			-
	Clade model (cetaceans)	−4594.72	p_0_ = 0.68694, p_1_ = 0.04973,p_2_ = 0.26333, branch type 0: ω_0_ = 0.02043,ω_1_ = 1.00000, ω_2_ = 0.19272, branch type 1: ω_0_ = 0.02043, ω_1_ = 1.00000, ω_2_ = 0.43113	(M1a vs. Clade Model) 104.1	<10^−16^	-
	**Branch-site models**					
	Model A	−4643.53	p_0_ = 0.74119, p_1_ = 0.09943, p_2_ = 0.14053,p_3_ = 0.01885, ω_0_ = 0.05388, ω_1_ = 1, ω_2_ = 1	(M1a vs Model A) 486	<10^−16^	-
	Null model A (ω = 1)	−4643.53	p_0_ = 0.62272, p_1_ = 0.08364, p_2_ = 0.25887, p_3_ = 0.03477, ω_0_ = 0.05392, ω_1_ = 1, ω_2_ = 1	(model A vs Null model A) 2	1	15, 27, 28, 101, 118, 140

a : P(ω>1)>0.95 is shown in bold.

**Table 2:** The pair-wise evolution rate (i.e. dN/dS) among cetacean Mbs using the maximum likelihood approach described in Methods section.

L_b_whale									
S_whale	0.2761								
P_s_whale	0.2259	0.2122							
M_whale	0.2647	0.2741	0.2735						
M_h_whale	0.2433	0.1950	0.1890	0.1754					
P_b_whale	0.3057	0.2324	0.2636	0.1566	0.2386				
Sei_whale	0.3469	0.2538	0.2832	0.1262	0.2173	0.001			
S_b_whale	0.1079	0.2641	0.2166	0.2796	0.2723	0.3261	0.3705		
Dolphin	0.2805	0.2536	0.2374	0.2096	0.3328	0.2865	0.2592	0.3176	
	L_b_whale	S_whale	P_s_whale	M_whale	M_h_whale	P_b_whale	Sei_whale	S_b_whale	Dolphin

**Table 3:** The pair-wise evolution rate (i.e. dN/dS) among primate Mbs using the maximum likelihood approach described in Methods section.

Human								
Chimpanzee	0.0312							
Macaque	0.0635	0.0860						
Gibbon	0.0532	0.0774	0.0738					
Marmoset	0.1272	0.1480	0.0647	0.1101				
Gorilla	0.0435	0.0941	0.0949	0.1053	0.1666			
Lemur	0.0487	0.0514	0.0566	0.0511	0.0537	0.0513		
Galago	0.0964	0.0900	0.0742	0.1138	0.0753	0.0911	0.0905	
	Human	Chimpanzee	Macaque	Gibbon	Marmoset	Gorilla	Lemur	Galago

**Figure 2:**
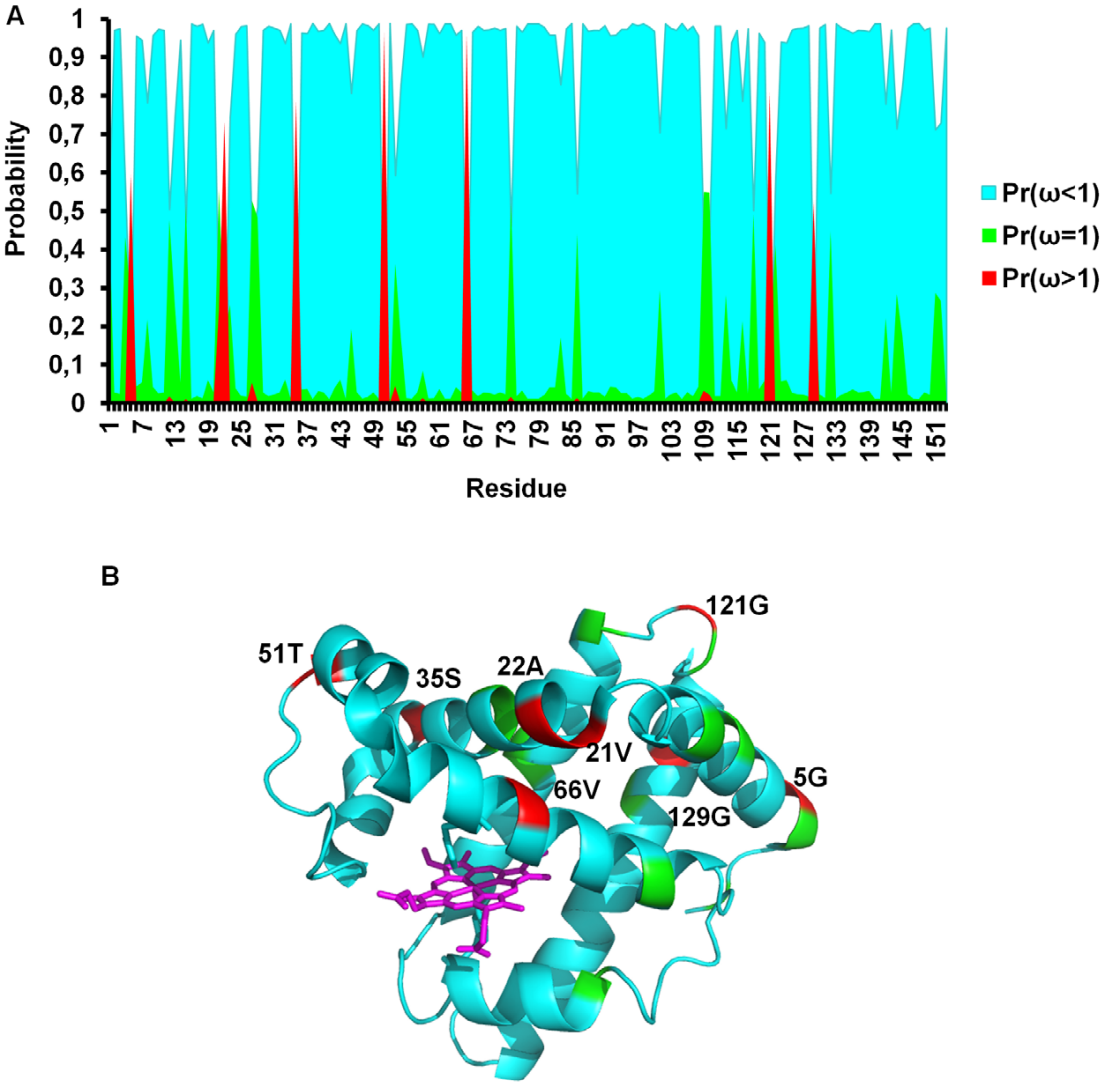
The Bayes empirical Bayes predictions for ω values for each site in cetacean Mb. A) For each residue p(ω<1), p(ω = 1) and p(ω>1) are shown in cyan, green and red respectively. Residues 5, 21, 22, 35, 51, 66, 121, and 129 have probabilities (ω>1)>0.5 with <ω> = 5.86 from the M8 model using the ML-estimated branch lengths under the M0 model. B) Crystal structure of sperm whale Mb taken from the protein data bank (ID = 1U7S) [32] with residues color coded by p(ω). The figure was created using PyMOL (http://www.pymol.org).

The higher rate of evolution in the cetacean clade could suggest accelerated evolution driven by positive selection of specific sites. To test this, we compared three site pair-models as M1–M2, M7–M8 and M8fix-M8 to identify sites under positive selection, as presented in Table 1 (see Methods section for details). From Table 1, the most stringent test (M8 vs. M8fix) indicated that seven sites (5, 22, 35, 51, 66, 121, and 129) are under positive selection with overall probabilities greater than 0.5 using the Bayes empirical Bayes (BEB) test [31]. Residue 21 was also detected to have a substantially high *dN/dS*, but its rate was not significantly greater than 1 and thus this residue was not detected by the BEB test. All eight sites are shown in Figure 2 with their posterior BEB probabilities using the M8 model, and with a mapping of sites onto the structure of sperm-whale Mb [32].

Table 1 also shows the results of a branch-site test of positive selection, model A, compared with M1a and the null model-A. Evolution rate (i.e. ω) was left to vary (model A) or fixed to 1 (null-model A) on the foreground tree with the marked branch leading to cetaceans (Figure 1A). The LRT was in this case not significant when model A was compared with its null model, but significant compared to model M1a.

### Ancestral state reconstruction and the evolution of stability

**Figure 3:**
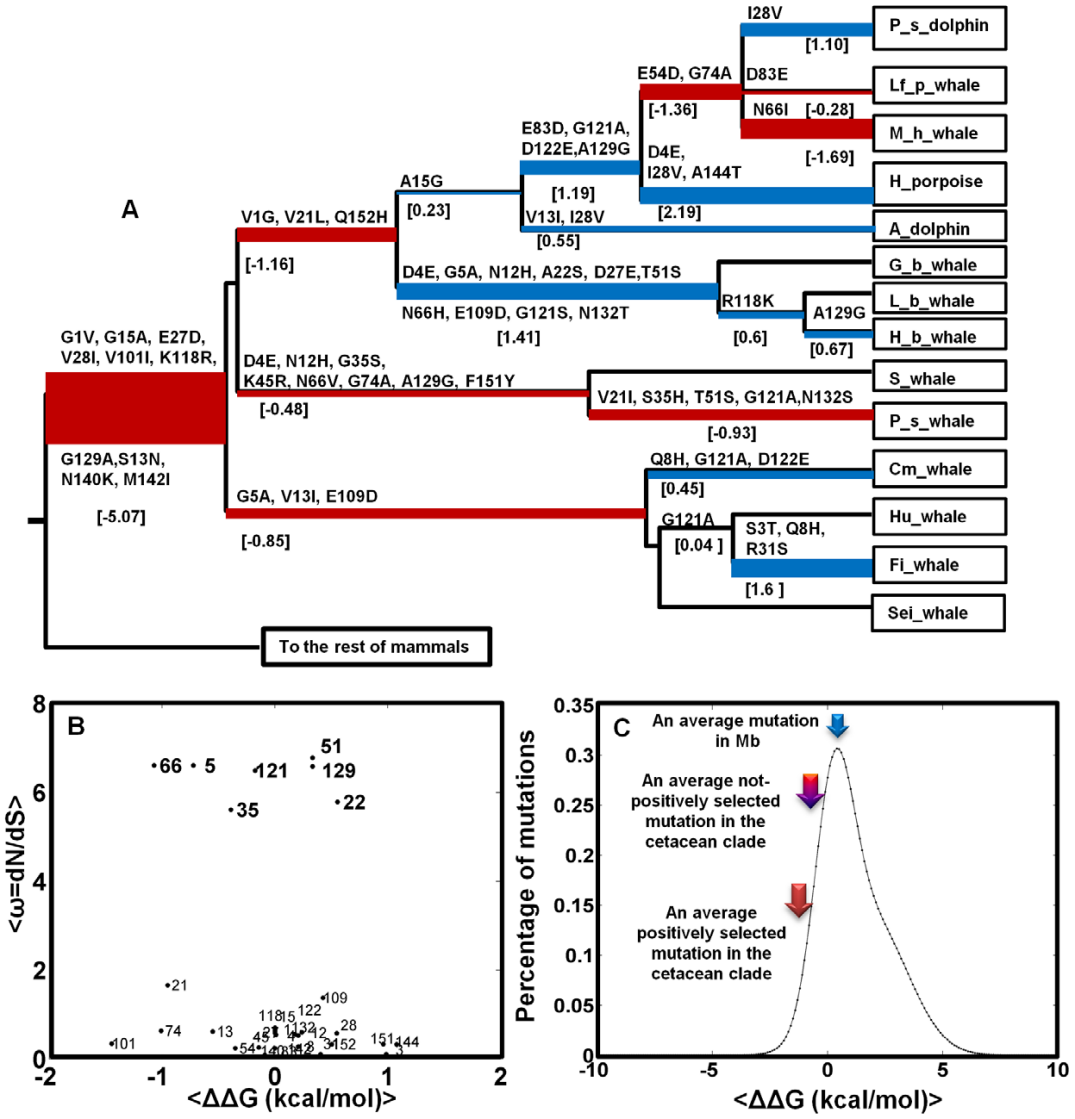
A) The Phylogenetic tree of cetacean Mb upon the divergence from terrestrial counterparts. Ancestral states were inferred using the maximum likelihood (ML) approach described in Methods [59]. Amino acid changes in each branch are shown with the respective changes in free energy of folding, ΔΔG in kcal/mol calculated from the FoldX force field [28]. Stabilization and destabilization is presented by red and blue colors respectively across the phylogeny, with branch height proportional to |ΔΔG| of that specific branch. B) The average ω = dN/dS for the variable sites in A from the M8 model is plotted versus the average ΔΔG of mutations in these sites. C) The distribution of mutational effects in Mb from [36] is shown with the solid black line where arrows show the average ΔΔG for an average mutation in Mb (∼1.22 kcal/mol), in the cetacean clade among not-positively selected mutations (∼0.06 kcal/mol) and, among the positively selected residues (∼−0.26 kcal/mol). The probability of stabilization caused by positive selection is ∼0.8.

To track the mutational pathways across different lineages of cetaceans, we constructed ancestral sequences as shown in Figure 3. Ancestral states were inferred using the large species tree in Figure 1B constructed from 82 Mb amino acid sequences, applying the Dayhoff substitution matrix allowing for among-site-rate-variation as explained in the Methods section. Overall probability of inference was 1 except in the sites 1, 13 and 28 where it is 0.5–0.9. In all of these sites, the alternative preferred amino acid is the initial mutated amino acid. Overall, our results did not encounter the problem of combinatorial ancestral characters that typically lead to non-unique reconstruction of ancestral sequences [33].

Using the FoldX algorithm, we computed the *ΔΔG* associated with the mutations in each branch of phylogeny as is shown in Figure 3. The overall stabilization or destabilization of each branch is depicted in red or blue, and the branch height is proportional to the absolute computed *ΔΔG* value of that specific branch. The overall stability increases in seven branches distributed from −0.3 to −5.1 kcal/mol.

Upon divergence of cetaceans from the rest of mammals, the most substantial increase of ∼5.1 kcal/mol was gained by mutations G15A, E27D, V28I, V101I, K118R, and G129A. From Table 1, the total ω is not significantly greater than 1, but this may be an unrealistically strict criterion for a small, highly constrained protein such as Mb, as evolutionary rate is strongly correlated to protein size due to the fraction of near-neutral sites increasing with size. Instead, LRT is significant when the branch-site test for positive selection (model A) is compared with the nearly neutral model (M1a), which indicates a higher ω in this first branch leading to cetaceans. In addition to positive selection under a new selection pressure (to be explained later, selection for a higher *C_Mb_* proportional to ADL, and additionally for folding stability), this might also be caused by relaxation of constraints (loss of selection pressure) [34]. Since the O_2_-binding affinity of Mb is nearly the same in all mammalian species (K_O2_ at 298 K and pH 7 of ∼0.8–1.2 µM^−1^), we conclude that the higher ω along this ancestral branch is consistent with positive selection under another arising selection pressure. As presented in Table 1, selection is further supported by the identified amino acid sites in the BEB test having high probabilities along this specific branch, and by the massive increase in the stability phenotype of ∼5 kcal/mol occurring during this branching. Altogether, these results suggest that the common ancestor of whales already possessed the new stability phenotype that will later be shown to imply that this ancestor was most likely a deep-diver, although our terminal nodes contain both terrestrial, shallow-, and deep-diving mammals.

After this early divergence that presumably established the majority of the new Mb stability, throughout the cetacean lineages, folding stability is seen to be maintained by fixation of several stabilizing mutations. From Figure 3A, the key mutations preserving this tendency are G5A, V13I, V21I, V21L, E27D, G35S, S35H, N66V, N66H, N66I, G74A, D83E, K118R, G121S, and G129A mutations. Eight of these mutations occur in the five sites 5, 35, 66, 121, and 129 which were detected by to be under positive selection. Thus, the insight from pure sequence-based maximum likelihood methods, amino acid substitution probabilities, and changes in biophysical stability as detected by structure-based approaches converge to the same interpretation of positive selection to obtain and maintain a higher Mb stability for the whales. As a further support for the link, G5A, G35S, and G129A mutations have been observed in more stable Mbs in comparative studies [14].

Figure 3B shows *dN/dS* values for the variable sites in the cetacean clade versus the inferred *ΔΔG* of the mutations. Four of the positively selected residues (i.e. residues 5, 35, 66, and 121) show an effect on folding stability >0.5 kcal/mol, with 5 and 66 being most significant, both towards stabilization (∼0.7 and ∼1.0 kcal/mol). Although the G129A mutation, which is fixated in the first branch leading to cetaceans (see Figure 3), is stabilizing (i.e. *ΔΔG* = −0.69 kcal/mol), it undergoes three inversions from Ala to Gly in the branches leading to sperm whales, beaked whales and the suborder of *Delphinidae*, which makes it net destabilizing when summing over occurrences, although this is less significant and could reflect a partial relaxation of stability selection. Insignificant destabilization is also observed in the residues 22 and 51 which will be discussed later.

Figure 3B and 3C show an interesting feature of the evolutionary dynamics of protein stability. As was recently shown by relating protein stability (i.e. *ΔG*) and evolution rate (i.e. *dN/dS*), proteins may evolve to a stability regime having a detailed balance between stabilizing and destabilizing mutations [35]. Without the stability effects of sites detected to be under positive selection, mutations are distributed nearly symmetrically in the *ΔΔG* vs. *dN/dS* scatter plot with an average mutation having *ΔΔG* = 0.1 kcal/mol. The average *ΔΔG* of an arising mutation in Mb is estimated to be ∼1.2 kcal/mol [36]. Together, these values suggest a balance between stabilizing and destabilizing mutations in the late branches of the cetacean clade.

Positive selection however shifts this balance by fixating stabilizing mutations such as G5A, G35S, S35H, N66V, N66H, N66I, G121S and G129A in the cetacean Mbs, providing a further stabilization of −1.7 kcal/mol for the whole clade and −4.4 kcal/mol when the branches leading to harbor porpoise and common minke whale are removed. These animals have *ΔG* similar to that of terrestrials both from experimental mutagenesis and stability measurements and from the FoldX computations. Also, they are shallow divers, consistent with their reduced *C_Mb_* (i.e. reduced need for a long ADL [6]), which might suggest that they are under less selection for stability (*vide infra*). Thus, after divergence towards the common deep-diving ancestor, positive selection still acted to maintain and purify Mb stability except in the mentioned case of apparent phenotype relaxation. The role of positive selection is also reflected in the probability of stabilization (i.e. *ΔΔG*<0 kcal/mol) conditional of positive selection, *pr (ΔΔG<0* | *ω>1)*, using the Bayes rule [37], being ∼0.80 (see Text S1 for details). Moreover, the average *ΔΔG* of positively selected residues is significantly less than that of non-positively selected residues with P-values of 0.0382 and 0.0456 using the two-sample t-test assuming unequal and equal variances in the two datasets, respectively.

**Figure 4:**
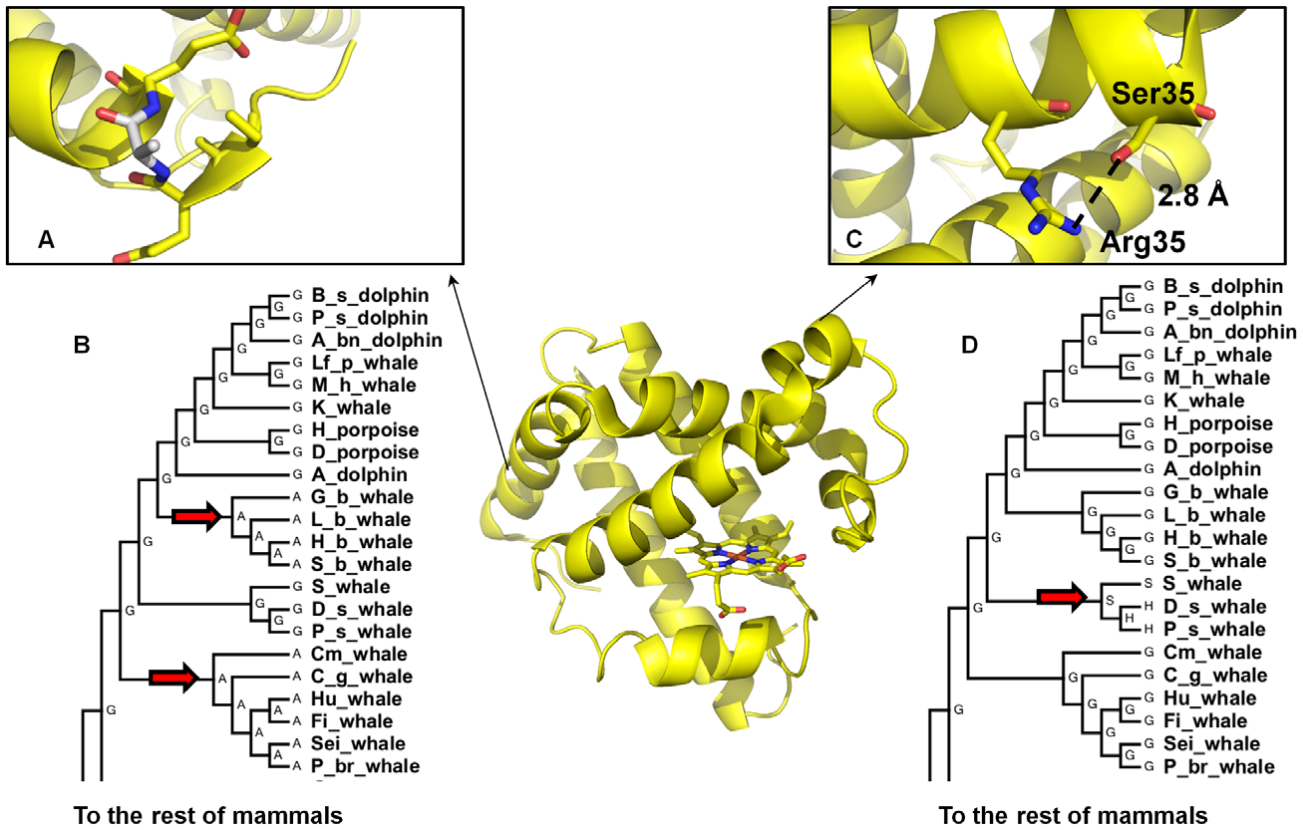
A) Ala at position 5 shown in the crystal structure of sperm whale Mb as preferred over Gly in two lineages within the cetacean phylogeny B) leading to Baleen whales and Beaked whales. C) Ser or His at position 35 is preferred over Gly for their ability to make a hydrogen bond with α-CO of Arg31 in the sperm whale clade of cetacean phylogeny D.

Among the seven positively selected sites, four sites display a mutation from Gly to Ala (1, 5, 121, and 129). Gly is known as a strong helix breaker and thus its replacement with Ala will strengthen the helix specifically in soluble proteins [38]. As is shown in Figure 4A and 4B, the G5A mutation is preferred in both Ziphidae (beaked whales) and Mysticeti (baleen whales) suborders of phylogeny. In position 66, a hydrophobic amino acid is stabilizing, confirmed by experimental measurements and most likely due to the hydrophobic effect (i.e. this mutation destabilizes the solvent exposed site in the unfolded protein relative to the folded protein). From Figure 4C, both Ser and His in position 35 can make a hydrogen bond to Arg31. The G35S/G35H mutations are selected in the two more stable physeter species (pygmy sperm whale and dwarf sperm whale) as is shown in Figure 4D. In position 51 which is a surface residue, a Thr to Ser mutation is preferred in two branches leading to beaked whales and to the more stable sperm whales. Both Thr and Ser have similar chemical properties and may form a hydrogen bond with αNH of residue 54 [14].

### Abundance and folding stability of cetacean Mbs correlate: Implications for fitness

So far we have shown that the systematic increase in folding stabilities of cetacean Mbs, partly known from experimental data and further elaborated by the FoldX calculations, is caused by positive selection in this clade of mammalian phylogeny. It is thus important to investigate the biological origin of the selection pressure driving this stabilization. Olson *et al.* has made the rationale for this increased stability as due to the sustained anaerobic and acidic conditions in the skeletal muscle of marine mammals [14], [16]. Since whales and seals experience prolonged dives, their Mbs have been suggested to be under selective pressure for increased resistance to unfolding during acidosis [14], [16].

This hypothesis is in contrast with several observations. First, marine mammals generally stay under aerobic metabolism due to the high cost of recovery after switch to anaerobic conditions [39]. The longest dives recorded for large whales such as blue and fin whales are much shorter than predicted the dive limits under aerobic conditions (ADL) [40]. In similar studies of sperm whales and seals, almost all the dives were found to not greatly exceed ADL [41], [42]. Second, the pH-fall in muscle and blood of seals after the long dives is reported to be less than one unit from its physiological value (∼7.5) which is too small to initiate unfolding [43]. These observations show that a switch to anaerobic metabolism and sustained acidosis in the muscle is less relevant for the diving patterns of marine mammals as observed in the wild [42].

**Figure 5:**
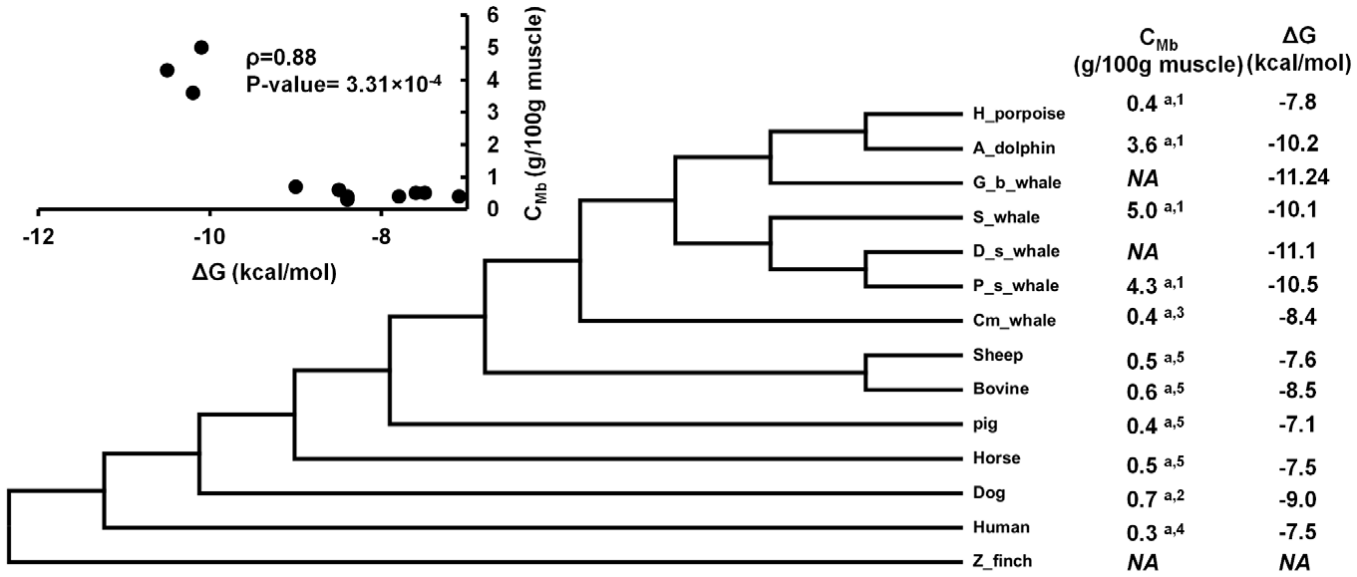
Divergence of cetaceans and the increase in Mb concentration by ∼10–20 fold. The experimental folding stability of apoMb is added to the difference in stability of holo and apoMb reported for horse heart Mb (2.7 kcal/mol). Stability is highly correlated with Mb concentration with correlation coefficient ρ = 0.88 and p-value = 0.000331. The Mb concentration has been measured in ^a^dorsi and in ^b^psaos muscle types. Data are taken from 1: [57], 2: [75], 3: [44], 4: [7] and 5: [76]. All the folding stabilities are taken from [14].

As seen in Figure 5, upon divergence of marine mammals, a ∼10–20 fold increase in Mb concentration (*C_Mb_*) is experimentally observed, which has been shown to be critical for O_2_ storage and diving capacity [6]. Moreover, the stability of Mb is also increased: For Pig, Horse, Sheep, Human, Bovine and Dog, *ΔG* of apoMb has been reported to be −4.4, −4.8, −4.9, −5.7, −5.8 and −6.3 kcal/mol [14], increasing to −5.1, −7.4, −7.5, −7.8, −8.4 and −8.7 in Dwarf sperm whale (*K. simus*), Pygmy sperm whale (*K. breviceps*), Sperm whale (*P. catadon*), Goose beak whale (*Z. cavirostris*), Dolphin (*Delphinus delphis*), and Minke whale (*B. acutorostrata*). The stability of holoMb is ∼2.7 kcal/mol higher than that of apoMb and this difference is assumed to be a constant, since residues in the heme pocket are conserved across all cetaceans [12], [14]. The average stability of holoMb is thus ∼−7 to −8 kcal/mol for terrestrial mammals and ∼−10 to −11 kcal/mol for cetaceans. More importantly, as shown in Figure 5, stability is highly correlated with the species-specific *C_M_*_b_ with a correlation coefficient ρ = 0.88 at the significance level <0.01. This correlation cannot be explained by adaptation to acidic conditions, because acidic robustness would not depend on protein abundance.

The *ΔG−C_Mb_* correlation is sensitive to various factors: First, *C_Mb_* varies somewhat among different muscle types in mammals. Swimming muscles in dolphins contains ∼82–86% of total Mb but constitute ∼75–80% of total muscle mass, compared to non-swimming muscles [44]. In humans, it is generally known that slow oxidative type I muscles contain more Mb than fast twitch type II muscles [45]. Second, Mb concentration is also age-dependent. Several studies of marine mammals suggest that skeletal muscle of pups have approximately 30% less Mb compared to adults [46], [47]. Despite these individual and tissue-wise variations in Mb expression, *C_Mb_* for marine mammals is still generally ∼10 fold higher than for terrestrial mammals [48].

### Evolution against burden of protein misfolding as *C_Mb_* increases

The correlation between protein folding stability and its expression level in the cell was recently proposed to be a consequence of protein misfolding prevention [17]. This hypothesis could explain the universal, strong anti-correlation between protein expression level and evolution rate (ER) in proteins, known as ER anti-correlation, i.e. highly expressed proteins are under stronger selection for stability to reduce the copy number of misfolded proteins [49]. While there may be many other explanations for the ER anti-correlation (i.e. the fitness impact, and hence conservation, of a protein would be proportional to its abundance regardless of the property selected for), the observation of a correlation between protein folding stability in Mb, as one of the most highly expressed mammalian proteins, and its abundance level in different organisms is the first, specific indication that stability as a protein phenotype may be the main property under selection in a real mammalian protein.

We propose that selection against unfolded protein is the cause of both the observed increased evolution rate (Table 1 and 2/3) and the higher stability of the cetacean Mbs. The increased evolution rate of cetacean Mbs with higher expression level seems at first to be in contrast with the average tendency of highly abundant proteins to evolve slowly [50], [51]. The explanation for this is most likely that highly expressed proteins that evolve slowly are normally close to equilibrium at their fitness optimum and under stronger selection for conserving stabilizing traits, whereas in the present specific evolutionary history, the increased evolutionary rate results from a divergence event where the higher abundance is established together with enhanced stability. This is fully consistent with our observed *C_Mb_*-stability correlation using available experimental data, with the dive depths of the respective animals, and with the observation of highest evolutionary rate during the first branching event where stability (and presumably *C_Mb_*) increased the most.

The present results thus also demonstrate how the evolution rate, dN/dS, of a single protein depends on a biophysical property such as in this case stability. Upon divergence to a new niche (deep-diving), the rate increased due to positive selection of new stabilizing mutations, but it is very conceivable that once the optimal stability has been obtained, fixation of new traits will also occur in cetacean Mbs, at least in so far as speciation is complete, which would reduce the rate of evolution as is partly seen in the latter part of the cetacean clade vs. the earlier part. Thus, our results are consistent with the general abundance-evolutionary rate anticorrelation but also suggest that the relation breaks down when highly expressed proteins undergo positive selection towards establishing new traits, leading to a speciation event of both higher evolutionary rate and higher abundance.

In this interpretation, upon the divergence of cetaceans from their terrestrial counterparts, the speciation towards deep divers quickly led to selection for higher *C_Mb_*, which for deep divers is almost proportional to ADL and by inference, fitness [6]. This early speciation led to an increased selection pressure acting to increase Mb stability in order to minimize the burden of misfolded Mbs within the cell. With a typical 10-fold increase in *C_Mb_*, an unchanged stability would increase the burden of unfolded Mb by 10-fold in cetaceans, but an average stability increase of ∼2 kcal/mol would change the folding equilibrium constant to keep the total copy number of unfolded Mb almost constant across lineages, implying that the burden would be checked in this way.

### Evolution of sites with no significant effect on stability

Among the significantly stabilizing mutations, 5, 35, and 66 were detected to be under positive selection with high posterior probabilities (p (ω>1)∼0.80–0.95). The remaining detected sites under positive selection were not significantly affecting stability as seen in Figure 3B. However, they might affect the protein in various other ways that also relate to the increased need for Mb and the adaptation of Mb-enriched deep-divers such as increased signalling requirements or structure preservation beyond thermodynamic stability, e.g. kinetic denucleation/unfolding prevention.

Notably, sites 22 and 51 are predicted to be destabilizing by FoldX in an agreement with previous comparative mutagenesis experiments [16]. Since both these surface residues are substituted for Ser, they may be involved in post translational modifications such as phosphorylation, although a physiological role phosphorylation is unknown [52]. In fact, both residues 22 and 51 are predicted to be phosphorylation sites in whale Mbs using the NetPhos 2.0 server (available at http://www.cbs.dtu.dk/services/NetPhos/) with high scores of 0.82 and 0.97, respectively (See Text S1). Moreover, residue 117 is also detected here as a phosphorylation site as proposed relevant for Beluga whale (*Delphinapterus leucas*) Mb [52]. This observation is consistent with previous studies in enzymes that gain-of-function mutations are on average destabilizing [53], but overall, positive selection still contributes to stability despite these marginally destabilizing sites.

### Concluding remarks

This work suggests that in an important real case of protein evolution, folding stability could be selected for in response to speciation in a new habitat: Our results suggest that the evolution of cetacean Mbs concurred with a divergence of one phenotype – stability – while oxygenation properties remained similar. Folding stability increased significantly (∼5.1 kcal/mol) due to the fixation of G15A, E27D, V28I, V101I, K118R, and G129A mutations. We have explained how and why increased Mb stability correlates with increased protein abundance during this evolutionary event, which probably involved substantial competition and speciation as niches were established in the diving regime.

The early, substantial increase in folding stability was accompanied by a significantly higher *dN/dS* in the first branch leading to cetaceans as judged from the comparison between the nearly neutral model (M1a) and the branch-site model of positive selection on this specific branch. This initial gain of folding stability was then later maintained through the fixation of G5A, V13I, V21L, V21I, V28I, G35S, S35H, N66V, N66I, G74A, V101I, K118R, G121A, and G129A mutations which compensate the deleterious effects of various destabilizing mutations possibly having marginally beneficial fitness effects relating to e.g. regulation. The full picture of these other functionalities would be a relevant focus area in future work.

Later in the clade, we have observed relaxation of the selection for stability. Notably, the common minke whale (*Balaenopetra acutorostrata*) and harbor porpoise (*Phocoenoides phocoena*) display *ΔG* and *C_Mb_* similar to terrestrial mammals with −8.4 and −7.8 kcal/mol and 0.37 and 0.40 gram per 100 g muscle, respectively. Given the linear effect of *C_Mb_* on ADL and by inference the action radius and fitness of the marine mammals [3], [6], This observation might be explained by the reduced oxygen consumption demands of both species during diving: Common minke whale is the smallest of the baleen whales with short dive times of ∼5–10 minutes [54] compared to sperm whales with an average dive time of ∼45 min [55]. Porpoises are also shallow divers (<50 m) with dive times less than two minutes [56]. Therefore, the selective pressure towards more (and more stable) Mb seems to be relaxed in these species if our mechanism is correct, explaining why shallow divers such as porpoises have reverted to less stable Mb. However, across the species, other factors, notably body mass reducing metabolic rate of the animal, also contribute to the total ADL [57], and future data on dive capacities vs. Mb stability would help to clarify the validity of the inferred mechanism.

While evolution is often interpreted as selection for new protein functionality [58], the evolution of cetacean Mbs described in this paper provides the first real example of protein stability being selected for as a consequence of protein abundance, using as control the terrestrials that have 10-fold less Mb. The mechanism by which evolution still acts on the cetacean Mbs, in addition to conservation of the heme pocket due to the reversible binding requirement [13], appears to be one of reducing the animal's burden of the more unfolded Mb copies in the muscle cells by increasing the selection for stability of the highly expressed protein. We suggest that this is the main explanation for the observed accelerated evolution in the cetacean clade.

## Methods

### Phylogenetic analysis and ancestral state reconstruction

The mammalian species tree was analyzed with the MEGA5 package [59] to select the best nucleotide/protein model with the lowest BIC scores, which was the Tamura-Nei92 and Dayhoff model allowing among-site-rate-variation (ASRV) sampled from a discrete gamma distribution with four categories (See Text S1 for details) [60]–[62]. To infer the ancestral sequences of the cetacean clade, branch lengths were first estimated using the Dayhoff model with ASRV, and the Bayesian posterior probabilities were calculated for each possible ancestral state for each node [63]. To explore the ancestral sequences inferred, we then used the maximum likelihood method [64] instead of the maximum parsimony (MP) approach due to the limitations of MP in dealing with branch lengths and possible uncertainties in the phylogeny [65].

New Mbs of any member of Ancodonta such as Hippos *(Hippopotamus)*, Camelidae and more species from Cetardiodactyla order such as Alpaca *(Vicugna vicugna)* could possibly resolve better the branch leading to cetaceans and thus provide a finer tree for investigating the episodic nature of *dN/dS* with respect to protein stability.

### Estimating evolution rate and detecting adaptive evolution

The pair-wise comparisons of Mb sequences of cetaceans and primates shown in Table 1 were estimated by the Maximum likelihood approach with codon models in CODEML program implemented in the PAML suite [66]. The equilibrium codon frequencies were estimated from the products of the average observed nucleotide frequencies in the three codon positions (F3X4 model).

To detect adaptive evolution, three codon-based models of nucleotide substitutions for the data [67] with the maximum likelihood inference were employed, first via “branch models” that allow the ω ratio (i.e. dN/dS) to vary among branches in the phylogeny [68]; M0 (one ω ratio for all lineages) and FR (one ω ratio for each branch), and second, via “site models” that allow the ω ratio to vary among codon sites within the sequence [69]. We used five different models referred to as M1 (nearly neutral), M2 (positive selection), M7 (beta), M8 (beta and ω), and M8fix (M8 with ω fixed at 1) [31]. The tree branch lengths were first estimated with the M0 model and were used in the more advanced codon models. We also used the site-models by estimating the branch lengths rather than taking their ML estimated values from the M0 model. With both approaches, the same sites were detected to be under positive selection with significant results in LRTs (see Table S3 in Text S1 for details). Positive selection in the specified residues was also robust to the use of gene tree instead of the organism tree (see Table S4 in Text S1 for details). Synonymous estimates in both marine and terrestrial mammals were less than 1.5 with the exception of one branch having ω = 1.56, and could thus be considered reliable. We ran the CODEML program several times with different initial values to prevent local optima in the Bayesian identification.

To compare the fit of nested models, classified as null and alternative models, the Likelihood Ratio Tests (LRT) was used [70]. Within a LRT test, twice the log-likelihood difference between two nested models has a chi-square distribution with a number of degrees of freedom equal to the free-parameter differences [71]. Different nested pairs of models were compared using the LRT such as branch models M0 versus FR, and Site models M1 versus M2, M7 versus M8, and M8fix versus M8. In cases where the LRT was significant, the Bayes empirical Bayes (BEB) method implemented for models M2 and M8 was employed to calculate the posterior probabilities for codon classes. A third class of LRT tests known as “branch-site” model that allow the ω ratio to vary among both sites and lineages [34] was also employed to infer positively selected sites in the ancestral branch leading to cetaceans. This branch-site test of positive selection was only used on the first branch leading to cetaceans to test the importance of this branching event in the overall divergence of cetaceans from terrestrials (shown with a black circle in Figure 1A). Any further statistical inference in the cetacean clade by detecting branches with high dN/dS values based on the free-ratio model should be corrected by the multiple-hypothesis corrections [72].

### Estimating effects of point mutations on folding stability

The initial 3D-structures used for calculating the stability of single point mutations were taken from the PDB structures of sperm whale Mb at 1.6 Å [32] and 1.4 Å resolution [73]. These structures were subject to the standard protocol of FoldX [28]. We validated the FoldX predicted *ΔΔG* values for both PDB structures against a set of experimentally reported Mb mutants. We then finally used the repaired PDB structure at 1.4 Å [73] which gave the strongest correlation between calculated and experimental *ΔΔG*s, for computing stabilities within the phylogeny. Individual mutations in the cetacean clade (Figure 3A) were built using “Build Model” command, and *ΔΔG* values were extracted from the FoldX output files. For both the validation set and mutations in Figure 3A, we repeated each mutation five times and took the average *ΔΔG* to reduce internal uncertainties of FoldX in estimating the stability effects of mutations, as recently recommended [74] (see Text S1 for details).

## Supporting Information

Text S1 Text S1 contains the following information: **Table S1:** Experimental and computed FoldX ΔΔG for a range of Mb mutations. The FoldX results (last two columns) are reported using two PDB structures: 1MBO and 1U7S. **Figure S1: Δ**ΔG values predicted by FoldX versus experimental ΔΔGs (kcal/mol) for the validation set (pdb = 1MBO). **Figure S2:** ΔΔG values predicted by FoldX versus experimental ΔΔGs (kcal/mol) for the validation set (pdb = 1U7S). **Table S2:** FoldX calculations for all mutations in the Cetacean clade using PDB structure 1U7S. Mutations in the sites detected to be under positive selection are shown in grey. **Table S3:** The best nucleotide and amino acid substitution models fitted to the data. **Table S4:** Results of amino acid substitution models for the whale clade. **Table S5:** Results of nucleotide substitution models for the whale clade. **Table S6:** Likelihood ratio tests for site models when branch lengths are estimated for each model rather than taking the ML-estimated branch lengths from the M0 model. LRT values are shown for M7 vs. M8 and M8 vs. M8fix. **Scheme S1:** Alignment for sperm whale, pig, bovine, dog, sheep, horse and human myoglobin (Mb) sequences. **Scheme S2:** The most probable cetacean ancestor with the complete phylogenetic tree (Figure 1-B), primate-rodent truncated tree, and only the cetacean clade. **Table S7:** LRT values for M7 vs. M8 and M8 vs. M8fix for the gene tree of cetaceans rather than using the species tree. **Table S8:** Species name and accession number of Mb sequences used in this study. The end of Text S1 contains CODEML and NetPhos Output.
(PDF)
