# Supplementary material for: Positively Selected Sites in Cetacean Myoglobins Contribute to Protein Stability
Source: PLoS Comput Biol. 2013 Mar 7;9(3):e1002929. doi: 10.1371/journal.pcbi.1002929 (PMC3591298; doi:10.1371/journal.pcbi.1002929)
Supplement: Text S1 — contains the following information: Table S1: Experimental and computed FoldX ΔΔG for a range of Mb mutations. The FoldX results (last two columns) are reported using two PDB structures: 1MBO and 1U7S. Figure S1: ΔΔG values predicted by FoldX versus experimental ΔΔGs (kcal/mol) for the validation set (pdb = 1MBO). Figure S2: ΔΔG values predicted by FoldX versus experimental ΔΔGs (kcal/mol) for the validation set (pdb = 1U7S). Table S2: FoldX calculations for all mutations in the Cetacean clade using PDB structure 1U7S. Mutations in the sites detected to be under positive selection are shown in grey. Table S3: The best nucleotide and amino acid substitution models fitted to the data. Table S4: Results of amino acid substitution models for the whale clade. Table S5: Results of nucleotide substitution models for the whale clade. Table S6: Likelihood ratio tests for site models when branch lengths are estimated for each model rather than taking the ML-estimated branch lengths from the M0 model. LRT values are shown for M7 vs. M8 and M8 vs. M8fix. Scheme S1: Alignment for sperm whale, pig, bovine, dog, sheep, horse and human myoglobin (Mb) sequences. Scheme S2: The most probable cetacean ancestor with the complete phylogenetic tree (Figure 1-B), primate-rodent truncated tree, and only the cetacean clade. Table S7: LRT values for M7 vs. M8 and M8 vs. M8fix for the gene tree of cetaceans rather than using the species tree. Table S8: Species name and accession number of Mb sequences used in this study. The end of Text S1 contains CODEML and NetPhos Output. (PDF) [file pcbi.1002929.s001.pdf]

Text S1

Positively selected sites in cetacean myoglobins contribute to protein Stability

Pouria Dasmeh[1,2], Adrian W.R Serohijos[2], Kasper P. Kepp[1*], Eugene I. Shakhnovich[2*]

[1]*Technical University of Denmark, DTU Chemistry, DK 2800 Kongens Lyngby, Denmark.*

[2] *Department of Chemistry and Chemical Biology, Harvard University, Cambridge, MA USA 02139.*

[*]*Correspondence:*

E.I.S. (shakhnovich@chemistry.harvard.edu)

K.P.K. (kpj@kemi.dtu.dk)

Table S1 Experimental and computed FoldX ΔΔG for a range of Mb mutations. The FoldX results (last two columns) are reported using two PDB structures: 1MBO and 1U7S.

Nr	**Reference**	Mutation	$\Delta\Delta G_{experimental}$ (unfolding)	pH	T	FoldX+ Repaired+1MBO (kcal/mol) [1]	FoldX+ Repaired+1u7s (kcal/mol) [2]
1	[3]	**WT**[a]	0	6	25	0	0
2	[3]	I28A	2.06	6	25	0.74	1.08
3	[3]	L29A	0.39	6	25	2.69	2.81
4	[3]	I30A	1.9	6	25	1.6	2.32
5	[3]	L32A	2.04	6	25	2.91	2.81
6	[4]	A130L	2.3	7.5	0	4.29	2.11
7	[4]	A130K	3.7	7.5	0	2.55	4.43
8	[4]	F123T	3.5	7.5	0	4.11	4.75
9	[5]	H24V	0.52	7	0	0.14	-1.23
10	[5]	H36Q	1.3	7	0	0.54	0.91
11	[5]	H48Q	0.62	7	0	-0.29	-0.42
12	[5]	H64Q	0.45	7	0	-0.29	-0.52
13	[5]	H82Q	0.05	7	0	2.24	1.34
14	[5]	H93G	-0.04	7	0	2.07	2.12
15	[5]	H97Q	0.11	7	0	-0.23	-0.26
16	[5]	H113Q	0.26	7	0	0.14	-0.48
17	[5]	H119F	0.68	7	0	-0.25	-0.5

a: Experimental ΔΔG values for the mutants are calculated from ΔG(mutant)- ΔG(WT) where ΔG(WT) is the respective WT free energy of unfolding for each group.

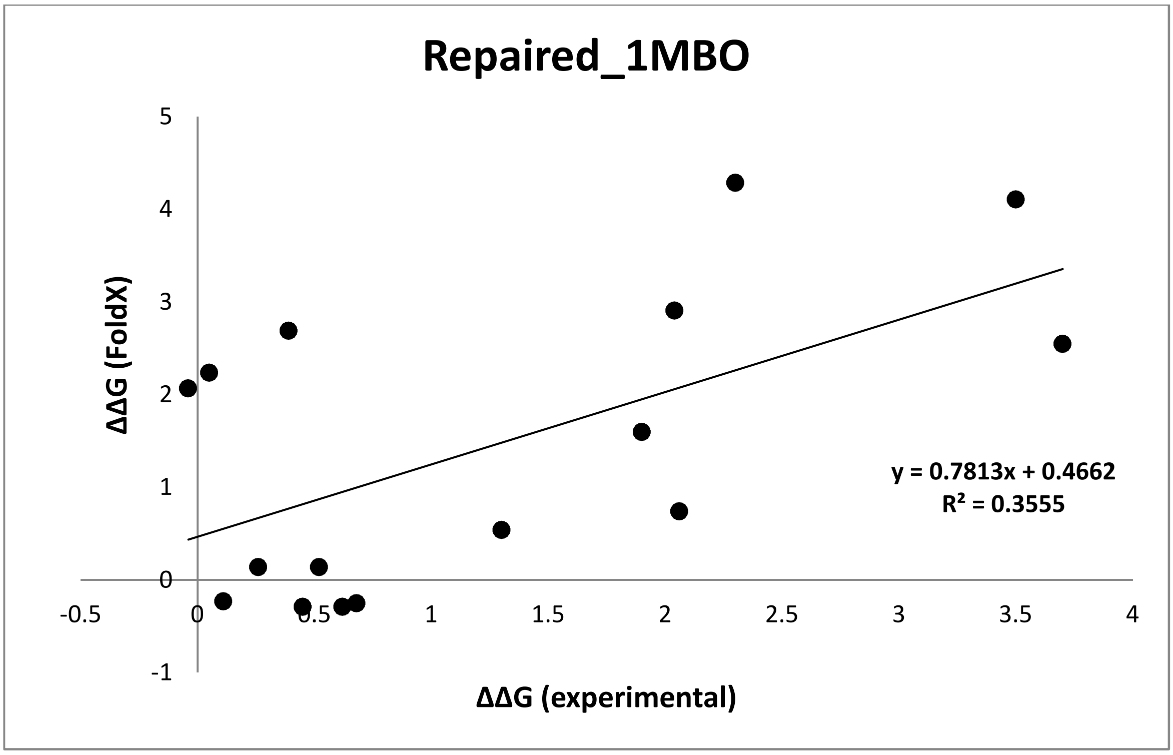

Figure S1 ΔΔG values predicted by FoldX versus experimental ΔΔGs (kcal/mol) for the validation set (pdb=1MBO).

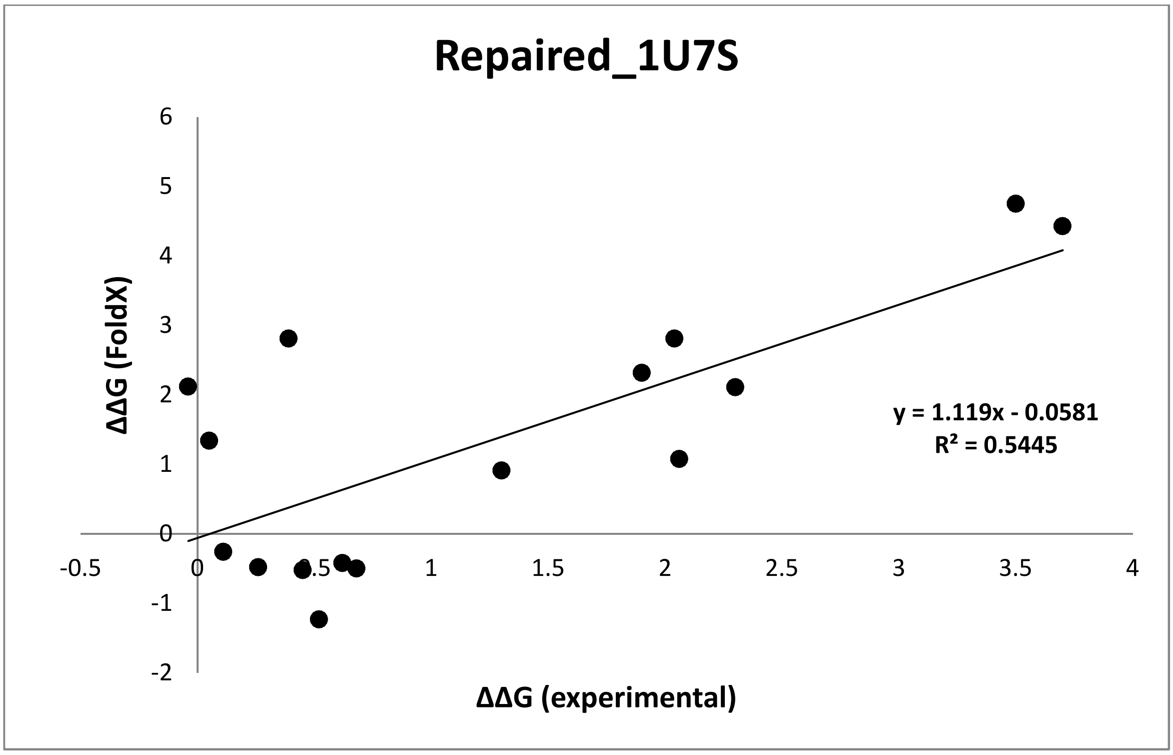

Figure S2 ΔΔG values predicted by FoldX versus experimental ΔΔGs (kcal/mol) for the validation set (pdb=1U7S).

Table S2 FoldX calculations for all mutations in the Cetacean clade using PDB structure 1U7S. Mutations in the sites detected to be under positive selection are shown in grey.

Mutation	ΔΔG	Mutation	ΔΔG
V1G	-0.444	N66V	-1.074
G1V	0.444	N66H	-0.444
S3T	0.988	N66I	-1.696
D4E	0.010	G74A	-1.008
G5A	-0.722	E83D	0.282
Q8H	0.208	D83E	-0.282
N12H	0.240	V101I	-1.446
V13I	-0.550	E109D	0.426
G15A	-0.230	K118R	-0.670
A15G	0.230	R118K	0.670
V21I	-0.678	G121A	0.038
V21L	-1.224	G121S	-1.032
A22S	0.556	D122E	0.208
E27D	-1.518	G129A	-0.670
D27E	1.518	A129G	0.670
V28I	-1.098	S132N	-0.007
I28V	1.098	N132S	0.007
R31S	0.404	N132T	0.530
G35S	-0.142	N140K	-0.090
S35H	-0.636	M142I	0.212
K45R	-0.142	A144T	1.080
T51S	0.334	F151Y	0.962
E54D	-0.352	Q152H	0.504

Probability of stabilization being conditional on the positive selection can be calculated as:

$$\Pr(\Delta\Delta G < 0|\omega > 1) = \frac{\Pr(\omega>1|\Delta\Delta G<0)\Pr(\Delta\Delta G<0)}{\Pr(\omega>1)} \quad \text{(S1)}$$

Overall, there are 63 different mutations in the whale, 26 mutations with ΔΔG < 0 and 17 mutations in the sites detected to be under positive selection with nine of them having ΔΔG < 0 (see Figure 3 in the main text). Equation S1 thus gives:

$$\Pr(\Delta\Delta G < 0|\omega > 1) = \frac{\left(\frac{9}{17}\right)\left(\frac{26}{63}\right)}{\left(\frac{17}{63}\right)} = 0.8090$$

Table S3 The best nucleotide and amino acid substitution models fitted to the data.

Model	#Param	BIC	AICc	lnL	Invariant	Gamma
Dayhoff+G	164	7024.6	5806.3	-2737	n/a	0.49985
Dayhoff+G+I	165	7032.9	5807.1	-2736.4	0.136066	0.67094
JTT+G	164	7040.8	5822.4	-2745.1	n/a	0.47808
JTT+G+I	165	7049.4	5823.6	-2744.6	0.145221	0.6543
WAG+G	164	7074.9	5856.5	-2762.1	n/a	0.45491
WAG+G+I	165	7083.2	5857.4	-2761.5	0.127089	0.58426
rtREV+G	164	7106.2	5887.8	-2777.8	n/a	0.44271
rtREV+G+I	165	7113.5	5887.8	-2776.7	0.155407	0.60074
Dayhoff+I	164	7163.5	5945.1	-2806.4	0.363738	n/a
JTT+I	164	7182.6	5964.2	-2816	0.374813	n/a
cpREV+G	164	7190.7	5972.4	-2820	n/a	0.38501
cpREV+G+I	165	7196.4	5970.6	-2818.1	0.209722	0.55634
JTT+G+F	183	7200.3	5841.3	-2735	n/a	0.48313
JTT+G+I+F	184	7208.6	5842.2	-2734.4	0.124288	0.61995
Dayhoff+G+F	183	7219.2	5860.3	-2744.5	n/a	0.49825
Dayhoff+G+I+F	184	7227.6	5861.2	-2743.9	0.117732	0.63561
WAG+I	164	7232.1	6013.7	-2840.7	0.374551	n/a
rtREV+I	164	7269.4	6051	-2859.4	0.374902	n/a
rtREV+G+F	183	7272	5913	-2770.8	n/a	0.44466
rtREV+G+I+F	184	7279.2	5912.9	-2769.7	0.149784	0.59206
WAG+G+F	183	7287.9	5928.9	-2778.8	n/a	0.45483
mtREV24+G	164	7289.7	6071.3	-2869.5	n/a	0.41432
WAG+G+I+F	184	7296.1	5929.7	-2778.2	0.120563	0.56934
mtREV24+G+I	165	7296.8	6071	-2868.3	0.146911	0.54439
mtREV24+G+F	183	7314.9	5956	-2792.3	n/a	0.43429
mtREV24+G+I+F	184	7323.5	5957.2	-2791.9	0.093653	0.5111
Dayhoff	163	7328.7	6117.7	-2893.7	n/a	n/a
JTT+I+F	183	7347.1	5988.1	-2808.4	0.368742	n/a
JTT	163	7361.5	6150.5	-2910.1	n/a	n/a
Dayhoff+I+F	183	7366.9	6007.9	-2818.3	0.358554	n/a
cpREV+I	164	7373.1	6154.7	-2911.2	0.395134	n/a
cpREV+G+F	183	7403.5	6044.5	-2836.6	n/a	0.39089
cpREV+G+I+F	184	7406	6039.6	-2833.1	0.266494	0.60489
WAG	163	7408.5	6197.5	-2933.6	n/a	n/a
rtREV+I+F	183	7434	6075	-2851.8	0.373158	n/a
WAG+I+F	183	7450.6	6091.7	-2860.2	0.369681	n/a
rtREV	163	7465.3	6254.3	-2962	n/a	n/a
mtREV24+I	164	7496.3	6277.9	-2972.8	0.362499	n/a
JTT+F	182	7508.4	6156.8	-2893.8	n/a	n/a
mtREV24+I+F	183	7509	6150.1	-2889.4	0.357606	n/a
Dayhoff+F	182	7522	6170.5	-2900.6	n/a	n/a
cpREV	163	7546.3	6335.3	-3002.5	n/a	n/a
cpREV+I+F	183	7576.7	6217.7	-2923.2	0.396104	n/a
WAG+F	182	7614.2	6262.6	-2946.7	n/a	n/a
rtREV+F	182	7618.3	6266.7	-2948.7	n/a	n/a
mtREV24+F	182	7675.5	6324	-2977.3	n/a	n/a
mtREV24	163	7723.3	6512.3	-3091	n/a	n/a
cpREV+F	182	7731.3	6379.7	-3005.2	n/a	n/a

Table S4 Results of amino acid substitution models for the whale clade.

Model	#Param	BIC	AICc	lnL
Dayhoff+G	18	1772.3	1676.6	-820.08
Dayhoff+G+I	19	1779.5	1678.6	-820.04
JTT+G	18	1784.5	1688.9	-826.22
JTT+G+I	19	1791.9	1690.9	-826.22
WAG+G	18	1796.7	1701	-832.27
rtREV+G	18	1799.9	1704.2	-833.87
WAG+G+I	19	1803.7	1702.7	-832.12
rtREV+G+I	19	1806.4	1705.5	-833.49
Dayhoff	17	1821.8	1731.4	-848.5
Dayhoff+I	18	1829.1	1733.4	-848.5
cpREV+G	18	1832.9	1737.3	-850.41
cpREV+I	18	1837	1741.4	-852.47
cpREV+G+I	19	1838.4	1737.4	-849.47
JTT	17	1847.7	1757.4	-861.48
JTT+I	18	1855.1	1759.4	-861.48
WAG	17	1864.5	1774.1	-869.87
Dayhoff+G+F	37	1868	1672.3	-798.2
WAG+I	18	1871.8	1776.2	-869.87
JTT+G+F	37	1873.7	1678	-801.06
Dayhoff+G+I+F	38	1875.3	1674.4	-798.2
rtREV	17	1877.4	1787	-876.3
JTT+G+I+F	38	1881	1680.1	-801.06
rtREV+I	18	1884.7	1789.1	-876.3
mtREV24+G	18	1886.1	1790.5	-877.01
rtREV+G+F	37	1886.6	1690.9	-807.53
mtREV24+G+I	19	1890.8	1789.9	-875.7
rtREV+G+I+F	38	1893.7	1692.8	-807.42
mtREV24+G+F	37	1900.7	1705	-814.58
WAG+G+F	37	1901.7	1706	-815.05
mtREV24+G+I+F	38	1902.7	1701.7	-811.89
WAG+G+I+F	38	1908.9	1708	-815.02
cpREV	17	1915.8	1825.4	-895.5
Dayhoff+F	36	1925.3	1734.8	-830.54
cpREV+G+F	37	1925.6	1729.9	-827.02
Dayhoff+I+F	37	1932.6	1736.9	-830.54
cpREV+G+I+F	38	1932.9	1732	-827.02
JTT+F	36	1937.1	1746.7	-836.46
JTT+I+F	37	1944.5	1748.8	-836.45
WAG+F	36	1968.8	1778.4	-852.3
WAG+I+F	37	1976.2	1780.5	-852.3
rtREV+F	36	1978.4	1787.9	-857.08
mtREV24	17	1979.5	1889.1	-927.35
mtREV24+F	36	1980.5	1790.1	-858.16
rtREV+I+F	37	1985.7	1790	-857.08
mtREV24+I	18	1986.8	1891.1	-927.34
mtREV24+I+F	37	1987.9	1792.2	-858.16
cpREV+F	36	1996.9	1806.5	-866.34
cpREV+I+F	37	2004.3	1808.6	-866.34

Table S5 Results of nucleotide substitution models for the whale clade.

Model	#Param	BIC	AICc	lnL	Invariant	Gamma	R
T92+G+I	21	2821.1	2686	-1321.9	0.581175	0.22705	1.4379
T92+G	20	2833.5	2704.8	-1332.3	n/a	0.09193	1.0517
K2+G+I	20	2834	2705.3	-1332.5	0.603384	0.2703	1.3252
HKY+G+I	23	2834.9	2686.9	-1320.3	0.578942	0.22564	1.4735
TN93+G+I	24	2839.4	2685	-1318.4	0.58886	0.22944	1.4997
K2+G	19	2839.6	2717.3	-1339.6	n/a	0.09298	0.9933
JC+G	18	2842.6	2726.7	-1345.3	n/a	0.09076	0.5
JC+I	18	2843.8	2727.9	-1345.9	0.788337	n/a	0.5
JC+G+I	19	2844.2	2721.9	-1341.9	0.596368	0.27056	0.5
HKY+G	22	2846.3	2704.7	-1330.3	n/a	0.09227	1.0712
HKY+I	22	2848.2	2706.6	-1331.2	0.791017	n/a	1.0625
TN93+G	23	2850.5	2702.5	-1328.1	n/a	0.10033	1.0612
GTR+G+I	27	2875.6	2701.9	-1323.8	0.613329	0.3932	1.1952
GTR+G	26	2875.8	2708.5	-1328.1	n/a	0.10037	1.0594
T92	19	2973.3	2851	-1406.4	n/a	n/a	0.916
K2	18	2974.2	2858.4	-1411.1	n/a	n/a	0.9147
JC	17	2977.7	2868.3	-1417.1	n/a	n/a	0.5
T92+I	20	2982.1	2853.4	-1406.6	0.00001	n/a	0.916
K2+I	19	2982.7	2860.4	-1411.1	0.00001	n/a	0.9147
HKY	21	2987.1	2851.9	-1404.9	n/a	n/a	0.9134
TN93	22	2992.5	2850.9	-1403.4	n/a	n/a	0.9175
TN93+I	23	2999.4	2851.5	-1402.6	0.00001	n/a	0.9173
GTR	25	3016	2855.2	-1402.5	n/a	n/a	0.9275
GTR+I	26	3022.9	2855.7	-1401.7	0.00001	n/a	0.9275

Table S6 Likelihood ratio tests for site models when branch lengths are estimated for each model rather than taking the ML-estimated branch lengths from the M0 model. LRT values are shown for M7 vs. M8 and M8 vs. M8fix.

Clades	Model	ln L	2Δl	P value	Positively selected sites (BEB: P(ω>1)>0.50)
**Cetaceans**	**Site models (number of parameters)**				
	M7	-1215.04			-
	M8	-1211.16	(M7 vs. M8) 7.76	0.0206	5, 22, 35, 51, 66, 121, 129
	M8fix	-1214.71	(M8fix vs M8) 7.1	0.007	

Scheme S1 Alignment for sperm whale, pig, bovine, dog, sheep, horse and human myoglobin (Mb) sequences.

CLUSTAL O(1.1.0) multiple sequence alignment

```
SP|sp|P02185|MYG_PHYMC|MYG_PHYMC MVLSEGEWQLVLHVWAKVEADVAGHGQDILIRLFKSHPETLEKFDRFKHLKTEAEMKASE 60
SP|sp|P02189|MYG_PIG|MYG_PIG     MGLSDGEWQLVLNVWGKVEADVAGHGQEVLIRLFKGHPETLEKFDKFKHLKSEDEMKASE 60
SP|sp|P02192|MYG_BOVIN|MYG_BOVIN MGLSDGEWQLVLNAWGKVEADVAGHGQEVLIRLFTGHPETLEKFDKFKHLKTEAEMKASE 60
SP|sp|P02159|MYG_LYCPI|MYG_LYCPI MGLSDGEWQIVLNIWGKVETDLAGHGQEVLIRLFKNHPETLDKFDKFKHLKTEDEMKGSE 60
SP|sp|P02190|MYG_SHEEP|MYG_SHEEP MGLSDGEWQLVLNAWGKVEADVAGHGQEVLIRLFTGHPETLEKFDKFKHLKTEAEMKASE 60
SP|sp|P68082|MYG_HORSE|MYG_HORSE MGLSDGEWQQVLNVWGKVEADIAGHGQEVLIRLFTGHPETLEKFDKFKHLKTEAEMKASE 60
SP|sp|P02144|MYG_HUMAN|MYG_HUMAN MGLSDGEWQLVLNVWGKVEADIPGHGQEVLIRLFKGHPETLEKFDKFKHLKSEDEMKASE 60
                                 * **:**** **. *.***:*: ****::*****. *****:***:*****:* ***.**

SP|sp|P02185|MYG_PHYMC|MYG_PHYMC DLKKHGVTVLTALGAILKKKGHHEAELKPLAQSHATKHKIPIKYLEFISEAIIHVLHSRH 120
SP|sp|P02189|MYG_PIG|MYG_PIG     DLKKHGNTVLTALGGILKKKGHHEAELTPLAQSHATKHKIPVKYLEFISEAIIQVLQSKH 120
SP|sp|P02192|MYG_BOVIN|MYG_BOVIN DLKKHGNTVLTALGGILKKKGHHEAEVKHLAESHANKHKIPVKYLEFISDAIIHVLHAKH 120
SP|sp|P02159|MYG_LYCPI|MYG_LYCPI DLKKHGNTVLTALGGILKKKGHHEAELKPLAQSHATKHKIPVKYLEFISDAIIQVLQNKH 120
SP|sp|P02190|MYG_SHEEP|MYG_SHEEP DLKKHGNTVLTALGGILKKKGHHEAEVKHLAESHANKHKIPVKYLEFISDAIIHVLHAKH 120
SP|sp|P68082|MYG_HORSE|MYG_HORSE DLKKHGTVVLTALGGILKKKGHHEAELKPLAQSHATKHKIPIKYLEFISDAIIHVLHSKH 120
SP|sp|P02144|MYG_HUMAN|MYG_HUMAN DLKKHGATVLTALGGILKKKGHHEAEIKPLAQSHATKHKIPVKYLEFISECIIQVLQSKH 120
                                 ****** .******.***********:.  **:***.*****:*******:.**:**: :*

SP|sp|P02185|MYG_PHYMC|MYG_PHYMC PGDFGADAQGAMNKALELFRKDIAAKYKELGYQG 154
SP|sp|P02189|MYG_PIG|MYG_PIG     PGDFGADAQGAMSKALELFRNDMAAKYKELGFQG 154
SP|sp|P02192|MYG_BOVIN|MYG_BOVIN PSDFGADAQAAMSKALELFRNDMAAQYKVLGFHG 154
SP|sp|P02159|MYG_LYCPI|MYG_LYCPI SGDFHADTEAAMKKALELFRNDIAAKYKELGFQG 154
SP|sp|P02190|MYG_SHEEP|MYG_SHEEP PSDFGADAQGAMSKALELFRNDMAAQYKVLGFQG 154
SP|sp|P68082|MYG_HORSE|MYG_HORSE PGDFGADAQGAMTKALELFRNDIAAKYKELGFQG 154
SP|sp|P02144|MYG_HUMAN|MYG_HUMAN PGDFGADAQGAMNKALELFRKDMASNYKELGFQG 154
                                 .** **::.**.*******:*:*::** **::*
```

Scheme S2 The most probable cetacean ancestor with the complete phylogenetic tree (Figure 1-B), primate-rodent truncated tree, and only the cetacean clade.

```
Truncated tree     MVLSDGEWQLVLNVWAKVEADVAGHGQDILIRLFKGHPETLEKFDKFKHLKTEAEMKASE 60
Cetacean clade     MVLSDAEWQLVLNIWAKVEADVAGHGQDILIRLFKGHPETLEKFDKFKHLKTEAEMKASE 60
Complete tree      MVLSDGEWQLVLNVWAKVEADVAGHGQDILIRLFKGHPETLEKFDKFKHLKTEAEMKASE 60
                   *****.*******:**********************************************

Truncated tree     DLKKHGNTVLTALGGILKKKGHHEAELKPLAQSHATKHKIPIKYLEFISEAIIHVLHSRH 120
Cetacen clade      DLKKHGNTVLTALGGILKKKGHHEAELKPLAQSHATKHKIPIKYLEFISDAIIHVLHSRH 120
Complete tree      DLKKHGNTVLTALGGILKKKGHHEAELKPLAQSHATKHKIPIKYLEFISEAIIHVLHSRH 120
                   ****************************************************:**********

Truncated tree     PGDFGADAQAAMNKALELFRKDIAAKYKELGFQG 154
Cetacean clade     PGDFGADAQAAMNKALELFRKDIAAKYKELGFQG 154
Complete tree      PGDFGADAQAAMNKALELFRKDIAAKYKELGFQG 154
                   ****************************************
```

Evaluating the robustness of positive selection with the gene-tree rather organism-tree for cetacean Mbs

To evaluate the robustness of positive selection with the gene-tree rather the species-tree, we have used the Maximum Likelihood method based on the Dayhoff matrix based model to make the phylogeny as shown in Figure 3. The bootstrap consensus tree inferred from 1000 replicates is taken to represent the evolutionary history of the taxa analyzed. Branches corresponding to partitions reproduced in less than 50% bootstrap replicates are collapsed. The percentage of replicate trees in which the associated taxa clustered together in the bootstrap test (1000 replicates) are shown next to the branches. A discrete Gamma distribution was used to model evolutionary rate differences among sites (4 categories (+*G*, parameter = 0.6640)). The tree is drawn to scale, with branch lengths measured in the number of substitutions per site. The analysis involved 10 amino acid sequences. There were a total of 154 positions in the final dataset. Evolutionary analyses were conducted in MEGA5.

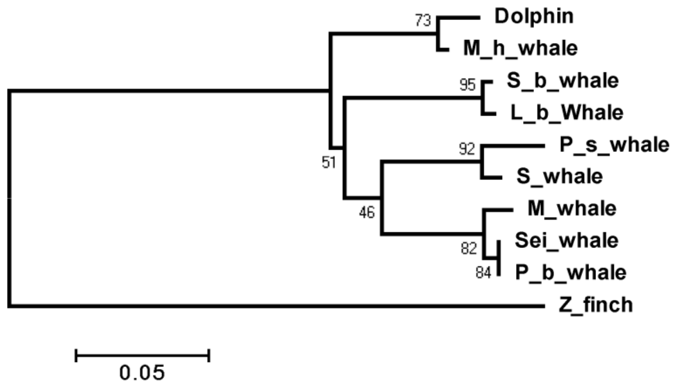

Figure 3. The gene-tree for the cetacean Mbs using the maximum likelihood estimation based on Dayhoff substitution model. Rate heterogeneity is allowed by using a discrete gamma distribution with four categories. The percentage of replicate trees in which the associated taxa clustered together in the bootstrap test (1000 replicates) are shown next to the branches.

Positive selection inferred in amino acid sites and judged by Likelihood ratio tests is also significant by using the gene-tree (see Table 1 below).

Table S7 LRT values for M7 vs. M8 and M8 vs. M8fix for the gene tree of cetaceans rather than using the species tree.

Model	ln L	2Δl	P-value	Positively selected sites (BEB: P(ω>1)>0.50)
M7	-1399.00			---
M8	-1391.65	(M7 vs. M8) 14.7	0.00064	5, 22, 35, 51, 66, 121, 129
M8fix	-1396.86	(M8fix vs M8) 10.42	0.0012	---

Table S8 Species name and accession number of Mb sequences used in this study.

Rank	Name in the phylogeny (full common name)	Species name	Accession number-Protein sequence[a]	Accession number-Nucleotide sequence
1	B_s_dolphin (Black sea dolphin)	Delphinus delphis	P68276	*NA*
2	P_s_dolphin (Pantropical spotted dolphin)	Stenella attenuata	Q0KIY7	BAF03580 [b]
3	A_bn_dolphin (Atlantic bottle-nosed dolphin)	Tursiops truncatus	P68279	*NA*
4	H_porpoise (Harbor porpoise)	Phocoenoides	P68278	*NA*
5	D_porpoise (Dall's porpoise)	Phocoenoides dalli dalli	P68277	*NA*
6	M_h_whale (Melon-headed whale)	Peponocephala electra	Q0KIY3	BAF03584 [b]
7	Lf_p_whale (Long-finned pilot whale)	Globicephala melas	P02174	*NA*
8	K_whale (Killer whale)	Orcinus orca	P02173	*NA*
9	A_dolphin (Amazon dolphin)	Inia geoffrensis	P02181	*NA*
10	S_b_whale (Stejneger's beaked whale)	Mesoplodon stejnegeri	Q0KIY0	BAF03587 [b]
11	H_b_whale (Hubb's beaked whale)	Mesoplodon carlhubbsi	P02183	*NA*
12	L_b_whale (Longman's beaked whale)	Indopacetus pacificus	Q0KIY9	BAF03578 [b]
13	G_b_whale (Goose-beaked whale)	Ziphius cavirostris	P02182	*NA*
14	P_b_whale (Pigmy Bryde's whale)	Balaenoptera edeni	Q0KIY2	BAF03585 [b]
15	S_whale (Sperm whale)	Physeter macrocephalus	P02185	BAF03579 [b]
16	P_s_whale (Pygmy sperm whale)	Kogia breviceps	Q0KIY5	BAF03582 [b]
17	D_s_whale (Dwarf sperm whale)	Kogia sima	P02184	*NA*
18	Hu_whale (Humpback whale)	Megaptera novaeangliae	P02178	*NA*
19	Fi_whale (Finback whale)	Balaenoptera physalus	P02180	*NA*
20	C_g_whale (California gray whale)	Eschrichtius gibbosus	P02177	*NA*
21	Sei_whale (Sei whale)	Balaenoptera borealis	Q0KIY1	BAF03586 [b]

NU=Not used, *NA*=Not Available, a: Taken from Uniprot database[6], b: Take from EMBL database[7], c:Taken from Ensembl genome browser[8].

Continued Table S8.

Rank	Name in the phylogeny (full common name)	Species name	Accession number-Protein sequence	Accession number-Nucleotide sequence
22	C_m_whale (Common minke whale)	Balaenoptera acutorostrata	P02179	BAF03583 [b]
23	Bovine (Bovine)	Bos taurus	P02192	BAA00311 [b]
24	A_bison (American bison)	Bison bison	P86873	*NA*
25	D_w_buffalo (Domestic water buffalo)	Bubalus bubalis	P84997	*NA*
26	W_yak (Wild yak)	Bos mutus grunniens	Q2MJN4	*NA*
27	Sheep (Sheep)	Ovis aries	P02190	ABJ97274 [b]
28	R_deer (Red deer)	Cervus elaphus	P02191	*NA*
29	Goat (Goat)	Capra hircus	B7U9B5	*NA*
30	Pig (Pig)	Sus scrofa	P02189	AAA31073 [b]
31	P_zebera (Plains zebra)	Equus burchelli	P68083	*NA*
32	Horse (Horse)	Equus caballus	P68082	NM_001164016.1 [c]
33	E_badger	Meles meles	P02157	*NA*
34	G_seal (Gray seal)	Halichoerus grypus	P68081	*NA*
35	H_seal (Harbor seal)	Phoca vitulina	P68080	*NA*
36	B_seal (Baikal seal)	Phoca sibirica	P30562	*NA*
37	Ca_sealion (California sealion)	Zalophus californianus	P02161	*NA*
38	E_r_otter (Eueopean river otter)	Lutra lutra	P11343	*NA*
39	Dog (Dog)	Canis familiaris	P63113	*NA*
40	Cat (Cat)	Felis catus	*NU*	ENSFCAT00000010057 [d]
41	A_w_dog (African wild dog)	Lycaon pictus	P02159	*NA*
42	C_fox (Cape fox)	Vulpes chama	P02160	*NA*
43	B_e_folx (Bat-eared fox)	Otocyon megalotis	P63114	*NA*
44	Microbat (Microbat)	Corynorhinus townsendii	*NU*	ENSMLUG00000013313 [c]
45	E_f_bat (Egyptian fruit bat)	Rousettus aegyptiacus	P02163	*NA*

Continued Table S8.

Rank	Name in the phylogeny (full common name)	Species name	Accession number-Protein sequence	Accession number-Nucleotide sequence
46	G_s_rat (Guaira spiny rat)	Proechimys guairae	P04249	*NA*
47	P_viscacha (Plains viscacha)	Lagostomus maximus	P04250	*NA*
48	N-gundi (Northern gundi)	Ctenodactylus gundi	P20856	*NA*
49	E_beaver (Eurasian beaver)	Castor fiber	P14396	*NA*
50	MBE_rat (Middle East blind mole rat)	Spalax ehrenbergi	P04248	*NA*
51	Muskrat (Muskrat)	Ondatra zibethicus	P32428	*NA*
52	Rat (Rat)	Rattus norvegicus	Q9QZ76	ENSDORG00000014500 [c]
53	K_rat (Kangaroo rat)	Dipodomys	*NU*	ENSDORG00000014500 [c]
54	Ginea_pig (Ginea pig)	Cavia porcellus	*NU*	ENSCPOG00000006864 [c]
55	Mouse (Mouse)	Mus musculus	P04247	ENSMUSG00000018893 [c]
56	S_a_pika (Southern American pika)	Ochotona princeps	P02171	*NA*
57	B_l_pika (Black-lipped pika)	Ochotona curzoniae	Q6PL31	*NA*
58	Rabbit (Rabbit)	Oryctolagus cuniculus	P02170	*NA*
59	Chimpanzee (Chimpanzee)	Pan troglodytes	P02145	ENSPTRG00000023553 [c]
60	Human (Human)	Homo sapiens	P02144	ENSG00000198125 [c]
61	M_gorilla (Mountain gorilla)	Gorilla gorilla beringei	P02147	ENSGGOG00000011478 [c]
62	B_orangutan (Bornean orangutan)	Pongo pygmaeus	P02148	*NA*
63	Siamang (Siamang)	Hylobates syndactylus	P62735	*NA*
64	A_gibbon (Agile gibbon)	Hylobates agilis	P62734	ENSNLEG00000014375 [c]
65	H_langur (Hanuman langur)	Semnopithecus entellus	P68085	*NA*
66	R_guenon (Red guenon)	Erythrocebus patas	P68086	*NA*
67	C_e_macaque (Crab-eating macaque)	Macaca fascicularis	P02150	ENSMMUG00000005034 [c]
68	O_baboon (Olive baboon)	Papio anubis	P68084	*NA*

Continued Table S8.

Rank	Name in the phylogeny (full common name)	Species name	Accession number-Protein sequence	Accession number-Nucleotide sequence
69	B_w_monkey (Brown woolly monkey)	Lagothrix lagotricha	P02154	*NA*
70	N_monkey (Night monkey)	Aotus trivirgatus	P02151	*NA*
71	Wte_marmoset (White-tufted-ear marmoset)	Callithrix jacchus	P02152	ENSCJAG00000000506 [c]
72	Cs_monkey (Common squirrel monkey)	Saimiri sciureus	P02155	*NA*
73	B_c_capuchin (Brown-capped capuchin)	Cebus apella	P02153	*NA*
74	G_galago (Greater galago)	Otolemur crassicaudatus	P02168	ENSOGAG00000005651 [c]
75	S_ioris (Slow loris)	Nycticebus coucang	P02167	*NA*
76	Potto (Potto)	Perodicticus potto edwarsi	P02166	*NA*
77	W_lemur (Weasel sportive lemur)	Lepilemur mustelinus	P02169	ENSMICG00000014107 [c]
78	T_shrew (Tree shrew)	Tupaia glis	P02165	ENSTBEG00000002813 [c]
79	Aardvark (Aardvark)	Orycteropus afer	P02164	
80	In_elephant (Indian elephant)	Elephas maximus	P02186	ENSLAFG00000023176 [c]
81	A_elephant (African elephant)	Loxodonta africana	P02187	*NA*
82	Hyrax (Hyrax)	Procavia capensis	*NU*	ENSPCAG00000003717 [c]
83	Na_opossum (North American opossum)	Didelphis marsupialis virginiana	P02193	*NA*
84	R_Kangaroo (Red kangaroo)	Macropus rufus	P02194	*NA*
85	A_echidna (Australian echidna)	Tachyglossus aculeatus aculeatus	P02195	*NA*

Continued Table S8.

Rank	Name in the phylogeny (full common name)	Species name	Accession number-Protein sequence	Accession number-Nucleotide sequence
86	D_platypus (Duckbill platypus)	Ornithorhynchus anatinus	P02196	ENSOANG00000010874 [c]
87	We_hedgehog (Western european hedgehog)	Erinaceus europaeus	P02156	ENSEEUG00000005138 [c]
88	Z_finch (Zebra finch)	Taeniopygia guttata	H0ZKN4	ENSTGUG00000010818 [c]

CODEML output for ML estimation of dN/dS for the mammalian tree.

TREE # 1: ((((((((((((6, 8), 1), (5, 9)), (2, 3)), (4, 7)), (20, (18, 31))), 19), (23, 24)), 33), 26), (((((21, 22), 28), 34), 29), ((16, 17), (14, (12, (13, (15, (10, 11)))))))), (25, 27)), 32, 30); MP score: 810

lnL(ntime: 65 np:130): -4872.649004 +0.000000

35..36 36..37 37..38 38..39 39..40 40..41 41..42 42..43 43..44 44..45 45..46 46..47 47..6 47..8 46..1 45..48 48..5 48..9 44..49 49..2 49..3 43..50 50..4 50..7 42..51 51..20 51..52 52..18 52..31 41..19 40..53 53..23 53..24 39..33 38..26 37..54 54..55 55..56 56..57 57..58 58..21 58..22 57..28 56..34 55..29 54..59 59..60 60..16 60..17 59..61 61..14 61..62 62..12 62..63 63..13 63..64 64..15 64..65 65..10 65..11 36..66 66..25 66..27 35..32 35..30

0.584809 0.074657 0.042898 0.000004 0.010941 0.034051 0.060302 0.110256 0.000004 0.013758 0.101162 0.000004 0.144838 0.007257 0.016031 0.090745 0.013375 0.019617 0.089965 0.013714 0.068115 0.053076 0.047203 0.000004 0.016442 0.136696 0.205366 0.072308 0.031778 0.294705 0.100081 0.271188 0.129317 0.429174 0.468055 0.000004 0.070835 0.048026 0.023908 0.409510 0.122394 0.107222 0.310555 0.403000 0.167701 0.000004 0.133405 0.153733 0.244236 0.133067 0.177681 0.062640 0.104159 0.040638 0.023365 0.006463 0.019474 0.008152 0.036874 0.014403 0.045353 0.274313 0.174368 0.316042 1.076215 0.030549 0.104180 0.082713 0.000100 999.000000 0.163035 0.048265 0.151570 0.000100 999.000000 0.623015 0.000100 0.397076 0.000100 0.133631 0.152928 0.183448 0.399032 0.358904 0.000100 0.265186 0.291258 0.137223 0.000100 0.137245 0.056099 0.157465 0.041776 0.147009 0.037426 0.107210 0.033873 0.328011 0.152701 0.034302 7.069095 0.086784 0.073461 999.000000 0.058117 0.078421 0.124204 0.053060 0.041973 0.021899 0.000100 0.032308 0.101873 0.104325 0.081870 0.122353 0.039095 0.014248 0.718144 0.000100 999.000000 0.096304 0.000100 0.000100 0.156876 0.255623 0.103774 0.084310 0.048824 0.080762

Note: Branch length is defined as number of nucleotide substitutions per codon (not per neucleotide site).

tree length = 8.45964

((((((((((((6: 0.144838, 8: 0.007257): 0.000004, 1: 0.016031): 0.101162, (5: 0.013375, 9: 0.019617): 0.090745): 0.013758, (2: 0.013714, 3: 0.068115): 0.089965): 0.000004, (4: 0.047203, 7: 0.000004): 0.053076): 0.110256, (20: 0.136696, (18: 0.072308, 31: 0.031778): 0.205366): 0.016442): 0.060302, 19: 0.294705): 0.034051, (23: 0.271188, 24: 0.129317): 0.100081): 0.010941, 33: 0.429174): 0.000004, 26: 0.468055): 0.042898, (((((21: 0.122394, 22: 0.107222): 0.409510, 28: 0.310555): 0.023908, 34: 0.403000): 0.048026, 29: 0.167701): 0.070835, ((16: 0.153733, 17: 0.244236): 0.133405, (14: 0.177681, (12: 0.104159, (13: 0.023365, (15: 0.019474, (10: 0.036874, 11: 0.014403): 0.008152): 0.006463): 0.040638): 0.062640): 0.133067): 0.000004): 0.000004): 0.074657, (25: 0.274313, 27: 0.174368): 0.045353): 0.584809, 32: 0.316042, 30: 1.076215);

((((((((((((P_b_whale: 0.144838, S_b_whale: 0.007257): 0.000004, L_b_whale: 0.016031): 0.101162, (M_h_whale: 0.013375, Dolphin: 0.019617): 0.090745): 0.013758, (S_whale: 0.013714, P_s_whale: 0.068115): 0.089965): 0.000004, (M_whale: 0.047203, Sei_whale: 0.000004): 0.053076): 0.110256, (Pig: 0.136696, (Sheep: 0.072308, Cow: 0.031778): 0.205366): 0.016442): 0.060302, Horse: 0.294705): 0.034051, (Cat: 0.271188, Dog: 0.129317): 0.100081): 0.010941, Microbat: 0.429174): 0.000004, Hedgehog: 0.468055): 0.042898, (((((Rat: 0.122394, Mouse: 0.107222): 0.409510, K_rat: 0.310555): 0.023908, Guinea_pig: 0.403000): 0.048026, Tree_shrew: 0.167701): 0.070835, ((Lemur: 0.153733, Galago: 0.244236): 0.133405, (Marmoset: 0.177681, (Macaque: 0.104159, (Gibbon: 0.023365, (Gorilla: 0.019474, (Human: 0.036874, Chimp: 0.014403): 0.008152): 0.006463): 0.040638): 0.062640): 0.133067): 0.000004): 0.000004): 0.074657, (Elephant: 0.274313, Hyrax: 0.174368): 0.045353): 0.584809, Platypus: 0.316042, Z_finch: 1.076215);

Detailed output identifying parameters

w (dN/dS) for branches: 0.03055 0.10418 0.08271 0.00010 999.00000 0.16303 0.04826 0.15157 0.00010 999.00000 0.62301 0.00010 0.39708 0.00010 0.13363 0.15293 0.18345 0.39903 0.35890 0.00010 0.26519 0.29126 0.13722 0.00010 0.13725 0.05610 0.15746 0.04178 0.14701 0.03743 0.10721 0.03387 0.32801 0.15270 0.03430 7.06910 0.08678 0.07346 999.00000 0.05812 0.07842 0.12420 0.05306 0.04197 0.02190 0.00010 0.03231 0.10187 0.10433 0.08187 0.12235 0.03909 0.01425 0.71814 0.00010 999.00000 0.09630 0.00010 0.00010 0.15688 0.25562 0.10377 0.08431 0.04882 0.08076

CODEML output for ML estimation of dN/dS for the whale clade of the mammalian tree.

TREE # 1: ((((1, 8), (5, 9)), (2, 3)), (6, (4, 7)), 10); MP score: 110
lnL(ntime: 17 np: 34): -1236.397819 +0.000000
11..12 12..13 13..14 14..1 14..8 13..15 15..5 15..9 12..16 16..2 16..3 11..17 17..6 17..18 18..4 18..7 11..10
0.000004 0.015419 0.108174 0.020475 0.000004 0.080726 0.013206 0.019818 0.099070 0.013827 0.068445 0.061192 0.000004 0.000004 0.040176 0.006770 0.338735 0.000100 999.000000 0.468419 0.092380 0.000100 0.215621 0.181910 0.403131 0.253993 0.000100 0.264662 0.239922 0.000100 0.000100 0.186734 0.000100 0.123082

Note: Branch length is defined as number of nucleotide substitutions per codon (not per neucleotide site).

tree length = 0.88605

((((1: 0.020475, 8: 0.000004): 0.108174, (5: 0.013206, 9: 0.019818): 0.080726): 0.015419, (2: 0.013827, 3: 0.068445): 0.099070): 0.000004, (6: 0.000004, (4: 0.040176, 7: 0.006770): 0.000004): 0.061192, 10: 0.338735);

((((L_b_Whale: 0.020475, S_b_whale: 0.000004): 0.108174, (M_h_whale: 0.013206, Dolphin: 0.019818): 0.080726): 0.015419, (S_whale: 0.013827, P_s_whale: 0.068445): 0.099070): 0.000004, (P_b_whale: 0.000004, (M_whale: 0.040176, Sei_whale: 0.006770): 0.000004): 0.061192, Human: 0.338735);

Detailed output identifying parameters

w (dN/dS) for branches: 0.00010 999.00000 0.46842 0.09238 0.00010 0.21562 0.18191 0.40313 0.25399 0.00010 0.26466 0.23992 0.00010 0.00010 0.18673 0.00010 0.12308

CODEML output for ML estimation of dN/dS for the terrestrial clade of the mammalian tree.

TREE # 1: (((((((((22, 9), 11), 10), (14, 15)), 24), 17), (((((13, 12), 19), 25), 20), ((8, 7), (5, (3, (4, (6, (2, 1)))))))), (16, 18)), 23, 21); MP score: 698

lnL(ntime: 0 np: 48): -4469.293861 +0.000000

2.132874 0.205803 0.177989 0.027162 0.000100 0.244110 0.023127 0.069289 0.239596 0.117675 0.052670 0.033534 0.098425 0.101625 0.071061 0.267009 0.295372 0.054535 0.176578 0.157013 0.107313 0.246148 0.090720 0.094597 0.141986 0.106614 0.065221 0.036812 0.000100 0.064889 0.117602 0.199427 0.134152 0.154916 0.035965 0.021182 0.414814 0.000100 999.000000 0.103703 0.000100 0.177970 0.000100 0.117072 0.130268 0.097307 0.055631 0.125422

tree length = 2.89227

(((((((((22: 0.014664, 9: 0.024835): 0.107857, 11: 0.033307): 0.024740, 10: 0.096967): 0.022258, (14: 0.061968, 15: 0.071931): 0.125947): 0.013695, 24: 0.159251): 0.020547, 17: 0.104168): 0.032203, (((((13: 0.059425, 12: 0.029436): 0.162509, 19: 0.077621): 0.017955, 25: 0.128501): 0.017460, 20: 0.044689): 0.011044, ((8: 0.125528, 7: 0.027376): 0.024531, (5: 0.079872, (3: 0.031680, (4: 0.006093, (6: 0.007188, (2: 0.006173, 1: 0.011604): 0.002617): 0.004348): 0.019334): 0.016000): 0.024475): 0.014916): 0.018029): 0.038019, (16: 0.112693, 18: 0.069882): 0.047069): 0.024887, 23: 0.161489, 21: 0.555492);

(((((((((Cow: 0.014664, Sheep: 0.024835): 0.107857, Pig: 0.033307): 0.024740, Horse: 0.096967): 0.022258, (Cat: 0.061968, Dog: 0.071931): 0.125947): 0.013695, Microbat: 0.159251): 0.020547, Hedgehog: 0.104168): 0.032203, (((((Mouse: 0.059425, Rat: 0.029436): 0.162509, K_rat: 0.077621): 0.017955, Guinea_pig: 0.128501): 0.017460, Tree_shrew: 0.044689): 0.011044, ((Galago: 0.125528, Lemur: 0.027376): 0.024531, (Marmoset: 0.079872, (Macaque: 0.031680, (Gibbon: 0.006093, (Gorilla: 0.007188, (Chimp: 0.006173, Human: 0.011604): 0.002617): 0.004348): 0.019334): 0.016000): 0.024475): 0.014916): 0.018029): 0.038019, (Elephant: 0.112693, Hyrax: 0.069882): 0.047069): 0.024887, Platypus: 0.161489, Z_finch: 0.555492);

Detailed output identifying parameters

kappa (ts/tv) = 2.13287

w (dN/dS) for branches: 0.20580 0.17799 0.02716 0.00010 0.24411 0.02313 0.06929 0.23960 0.11767 0.05267 0.03353 0.09843 0.10162 0.07106 0.26701 0.29537 0.05454 0.17658 0.15701 0.10731 0.24615 0.09072 0.09460 0.14199 0.10661 0.06522 0.03681 0.00010 0.06489 0.11760 0.19943 0.13415 0.15492 0.03597 0.02118 0.41481 0.00010 999.00000 0.10370 0.00010 0.17797 0.00010 0.11707 0.13027 0.09731 0.05563 0.12542

NetPhos 2.0 prediction results.

154 L_b_Whale
MGLSEAEWQLVLHVWAKVEADLSGHGQEILIRLFKGHPETLEKFDKFKHLKSEAEMKASEDLKKHGH
TVLTALGGILKKK 80
GHHEAELKPLAQSHATKHKIPIKYLEFISDAIIHVLHSKHPSDFGADAQAAMTKALELFRKDIAAKYKEL
GFHG 160
......................S............................S............................ 80
...................................S................................... 160

Phosphorylation sites predicted: Ser: 3 Thr: 0 Tyr: 0

Serine predictions

Name	Pos	Context	Score	Pred
L_b_Whale	4	-MGLSEAEW	0.008	.
L_b_Whale	23	EADLSGHGQ	0.829	*S*
L_b_Whale	52	KHLKSEAEM	0.975	*S*
L_b_Whale	59	EMKASEDLK	0.047	.
L_b_Whale	93	PLAQSHATK	0.018	.
L_b_Whale	109	LEFISDAII	0.003	.
L_b_Whale	118	HVLHSKHPS	0.973	*S*
L_b_Whale	122	SKHPSDFGA	0.249	.

Threonine predictions

Name	Pos	Context	Score	Pred
L_b_Whale	40	GHPETLEKF	0.361	.
L_b_Whale	68	KHGHTVLTA	0.013	.
L_b_Whale	71	HTVLTALGG	0.141	.
L_b_Whale	96	QSHATKHKI	0.076	.
L_b_Whale	133	QAAMTKALE	0.296	.

Tyrosine predictions

Name	Pos	Context	Score	Pred
L_b_Whale	104	IPIKYLEFI	0.025	.
L_b_Whale	147	IAAKYKELG	0.467	.

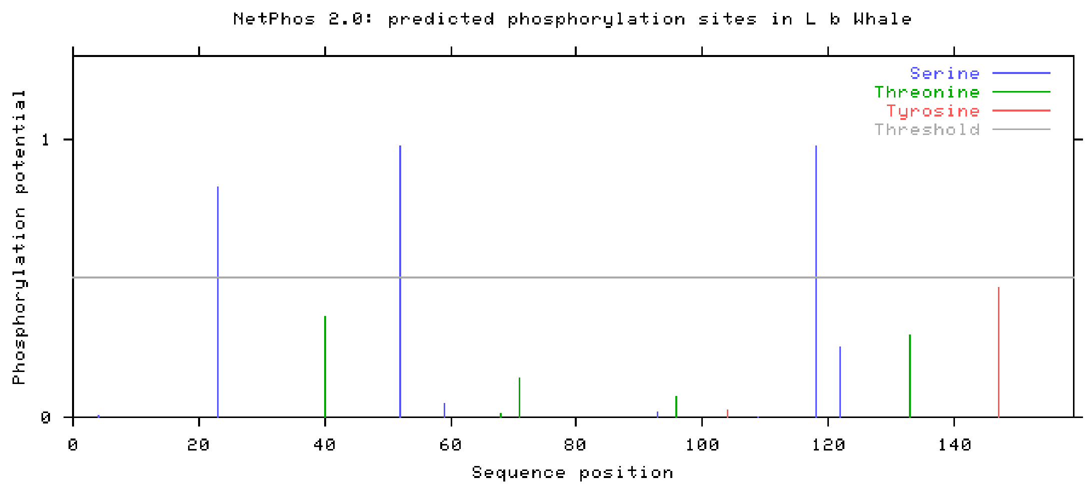

154 S_b_whale
MGLSEAEWQLVLHVWAKVEADLSGHGQEILIRLFKGHPETLEKFDKFKHLKSEAEMKASEDLKKHGH
TVLTALGGILKKK 80
GHHEAELKPLAQSHATKHKIPIKYLEFISDAIIHVLHSKHPSDFGADAQGAMTKALELFRKDIAAKYKEL
GFHG 160
......................S............................S............................ 80
....................................S.................................... 160

Phosphorylation sites predicted: Ser: 3 Thr: 0 Tyr: 0

Serine predictions

Name	Pos	Context	Score	Pred
S_b_whale	4	-MGLSEAEW	0.008	.
S_b_whale	23	EADLSGHGQ	0.829	*S*
S_b_whale	52	KHLKSEAEM	0.975	*S*
S_b_whale	59	EMKASEDLK	0.047	.
S_b_whale	93	PLAQSHATK	0.018	.
S_b_whale	109	LEFISDAII	0.003	.
S_b_whale	118	HVLHSKHPS	0.973	*S*
S_b_whale	122	SKHPSDFGA	0.249	.

Threonine predictions

Name	Pos	Context	Score	Pred
S_b_whale	40	GHPETLEKF	0.361	.
S_b_whale	68	KHGHTVLTA	0.013	.
S_b_whale	71	HTVLTALGG	0.141	.
S_b_whale	96	QSHATKHKI	0.076	.
S_b_whale	133	QGAMTKALE	0.296	.

Tyrosine predictions

Name	Pos	Context	Score	Pred
S_b_whale	104	IPIKYLEFI	0.025	.
S_b_whale	147	IAAKYKELG	0.467	.

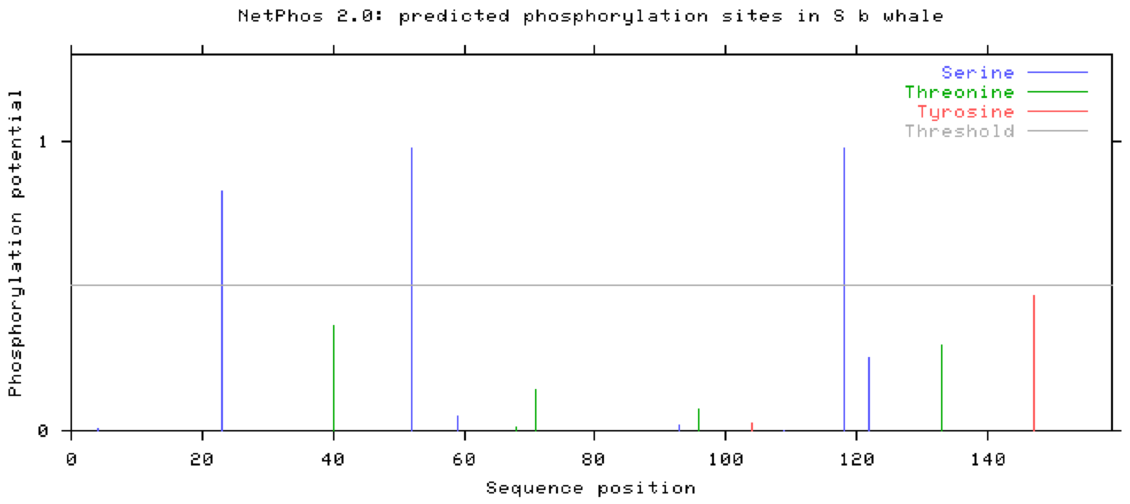

References

[1] Phillips SEV (1980) Structure and refinement of oxymyoglobin at 1.6 Å resolutions. J Mol Biol 142: 531-554.

[2] Kondrashov DA, Zhang W, Aranda IV R, Stec B, Phillips GN (2008) Sampling of the native conformational ensemble of myoglobin via structures in different crystalline environments. Proteins Struct Funct Bioinf 70:353–362.

[3] Nishimura C, Wright PE, Dyson HJ (2003) Role of the B helix in early folding events in apomyoglobin: evidence from site-directed mutagenesis for native-like long range interactions. J Mol Biol 334: 293–307.

[4] Hughson FM, Barrick D, Baldwin RL (1991) Probing the stability of a partly folded apomyoglobin intermediate by site-directed mutagenesis. Biochemistry 30: 4113-41 18.

[5] Barrick D, Hughson FM, Baldwin RL (1994) Molecular mechanisms of acid denaturation. The role of histidine residues in the partial unfolding of apomyoglobin. J Mol Biol 237:588–601.

[6] UniProt Consortium (2011) Ongoing and future developments at the Universal Protein Resource. Nucleic Acids Res 39, D214–D219.

[7] Kanz C, Aldebert P, Althorpe N, Baker W, Baldwin A et al. (2005) The EMBL Nucleotide Sequence Database. Nucleic Acids Res. 33:D29–D33.

[8] Hubbard TJ, Aken BL, Ayling S, Ballester B, Beal K ET AL. (2009) Ensembl 2009, Nucleic Acids Res. 2009; 37:D690–D697.
